# Lamellipodia-based migrations of larval epithelial cells are required for normal closure of the adult epidermis of *Drosophila*

**DOI:** 10.1016/j.ydbio.2011.12.033

**Published:** 2012-03-01

**Authors:** Marcus Bischoff

**Affiliations:** University of Cambridge, Department of Zoology, Downing Street, CB2 3EJ Cambridge, UK

**Keywords:** *In vivo* imaging, morphogenesis, *Drosophila* abdomen, Cell migration, Planar cell polarity, Apical constriction

## Abstract

Cell migrations are an important feature of animal development. They are, furthermore, essential to wound healing and tumour progression. Despite recent progress, it is still mysterious how cell migration is spatially and temporally regulated during morphogenesis and how cell migration is coordinated with other cellular behaviours to shape tissues and organs. The formation of the abdominal epithelium of *Drosophila* during metamorphosis provides an attractive system to study morphogenesis. Here, the diploid adult histoblasts replace the polyploid larval epithelial cells (LECs). Using *in vivo* 4D microscopy, I show that, besides apical constriction and apoptosis, the LECs undergo extensive coordinated migrations. The migrations follow a transition from a stationary (epithelial) to a migratory mode. The migratory behaviour is stimulated by autocrine Dpp signalling. Directed apical lamellipodia-like protrusions propel the cells. Initially, planar cell polarity determines the orientation of LEC migration. While LECs are migrating they also constrict apically, and changes in activity of the small GTPase Rho1 can favour one behaviour over the other. This study shows that the LECs play a more active role in morphogenesis than previously thought, with their migrations contributing to abdominal closure. It furthermore provides insights into how the migratory behaviour of cells is regulated during morphogenesis.

## Highlights

► The larval epithelial cells (LECs) undergo extensive coordinated migrations. ► These migrations follow a transition from a stationary to a migratory mode. ► Directed apical lamellipodia-like protrusions propel the cells. ► LEC migration is regulated by Dpp signalling, planar cell polarity and Rho1. ► LEC migration contributes to the morphogenesis of the adult abdominal epidermis.

## Introduction

Cell migrations are an important aspect of animal development (Montell, 1999). They are crucial to position cells during morphogenesis, where they also have to be coordinated with other cellular behaviours such as shape changes and divisions to form tissues and organs (Bischoff and Cseresnyes, 2009; Butler et al., 2009; Fernandez et al., 2007; Gong et al., 2004). Despite recent progress, it is still mysterious what regulates cell migration to ensure that cells migrate at the right time to the correct position and how the coordination of cell migration with other cell behaviours is achieved.

Moving cells are often of epithelial origin. To become mobile, these cells have to undergo a transition from a stationary to a migratory mode. During such epithelial to mesenchymal transitions (EMTs), cells lose epithelial characteristics, such as cell adhesion, and gain mesenchymal characteristics, such as becoming migratory (Baum et al., 2008; Huber et al., 2005; Thiery et al., 2009). Examples of such processes include *Drosophila* border cell migration (Niewiadomska et al., 1999) and gastrulation (Leptin, 1999), zebrafish lateral line migration (Haas and Gilmour, 2006) and neural crest migration in vertebrates (Matthews et al., 2008). Migration of epithelial cells also contributes to the closure of wounds (Yan et al., 2010) and to tumour progression when cancer cells invade surrounding tissues (Thiery, 2002).

The metamorphosis of the abdominal epithelium of *Drosophila* provides an attractive system to study the migration of epithelial cells. Here, the diploid adult histoblasts replace the polyploid larval epithelial cells (LECs) (Supplementary Movie 1) (Bischoff and Cseresnyes, 2009; Madhavan and Madhavan, 1980; Ninov et al., 2007). While the histoblasts divide and migrate towards the midline, the LECs constrict apically, leave the epithelium (delaminate) and die. This is concurrent with the notion that the larval tissue has to be removed to generate space for the adult tissue (Ninov et al., 2007, 2010). The mechanisms that drive morphogenesis of the adult epidermis are, however, still elusive.

We have previously shown that the LECs relocate dorsally before they die (Bischoff and Cseresnyes, 2009), which suggested that the LECs might be pushed by the histoblasts. Here I analyse the behaviour of the LECs in detail using *in vivo* 4D microscopy (Bischoff and Cseresnyes, 2009; Schnabel et al., 1997). I show that the LECs undergo extensive coordinated migrations, which are propelled by apical lamellipodia-like protrusions. These migrations are well suited to study how migratory behaviour is regulated during different phases of morphogenesis. Prior to migration, the LECs undergo a transition from stationary to migratory behaviour. The migratory behaviour is stimulated by autocrine Decapentaplegic (Dpp) signalling. Initially, the migrations are oriented posteriorly, and this depends on the planar polarity of the epithelium. Eventually, the LECs move dorsally, while also constricting apically. Altering levels of the small GTPase Rho1 can favour one behaviour over the other — Rho1 activation induces constriction, whereas its down-regulation increases migratory behaviour. Overall my data show that, together with apical constriction, the coordinated migration of LECs is required for normal closure of the adult epithelium. Thus, the LECs play a more active role in morphogenesis than previously thought.

## Materials and methods

### Fly stocks

FlyBase (Tweedie et al., 2009) entries of the used transgenes are as follows:

*en.Gal4*: *Scer/Gal4*^*en-e16E*^*, hh.Gal4*: *Scer/Gal4*^*hh-Gal4*^, *H2AvGFP*: *His2Av*^*T:Avic\GFP-S65T*^, *DE-cadherin::GFP*: *shg*^*Ubi-p63E.T:Avic\GFP-rs*^, *UAS.RFP: Disc\RFP*^*Scer\UAS.cWa*^, *UAS.GFP-actin*: *Act5C*^*Scer\UAS.T:Avic\GFP*^, *UAS.gma*: *Moe*^*Scer\UAS.T:Avic\GFP-S65T*^, *UAS.ectoDs*: *ds*^*ecto.Scer\UAS*^, *UAS.p35*: *BacA\p35*^*Scer\UAS.cHa*^, *UAS.DIAP1*: *th*^*Scer\UAS.T:Ivir\HA1*^, *UAS.DIAP2*: *lap2*^*Scer\UAS.cWa*^, *UAS.rho*^*V14*^: *Rho1*^*V14.Scer\UAS*^*, UAS.rho*^*N19*^: *Rho1*^*N19.Scer/UAS*^, *UAS.DCR2*: *Dcr-2*^*Scer/UAS.cDa*^, *UAS.tkv*^*DN*^: tkv^1ΔGSK.Scer/UAS^, *UAS.tkv*^*Q-D*^: *tkv*^*QD.Scer\UAS*^, *UAS.dad*: *dad*^*Scer/UAS.cTa*^, *UAS.pio-RNAi*: *pio*^*KK112233*^, *UAS.ds-RNAi*: *ds*^*GD14350*^, *UAS.rho1-RNAi*: *rho1*^*GD4726*^, *UAS.tkv-RNAi*: *tkv*^*GD2549*^, *UAS.dpp-RNAi*: *dpp*^*JF01371*^, *tub-FRT-CD2-FRT-Gal4*: *Rnor\CD2*^*A902*^, *UAS.mCD8-GFP*: *Mmus/Cd8a*^*Scer\UAS.T:Avic/GFP*^, *UAS.mCD8-RFP*: *Mmus/Cd8a*^*Scer\UAS.T:Disc/RFP-mRFP*^, *UAS.myr-RFP*: *Disc/RFP*^*Scer/UAS.T:Myr1*^, *UAS.FLP*: *FLP1*^*Scer\UAS.cDa*^.

### Expression of UAS-transgenes in LECs

Overexpression of *UAS*-transgenes in the LECs was achieved by using the FLP-out technique (Struhl and Basler, 1993). FLP-out clones were induced by heat-shocking third instar larvae for 6 to 10 min at 35–37 °C. This led to FLP-out events in the polyploid LECs but rarely in the diploid histoblasts (Ninov et al., 2007). Thus, most LECs but only few histoblasts expressed the UAS-transgenes. Additional expression of *UAS.FLP* increased the recombination events in the polyploid cells, thus increasing expression levels. After heat-shock, flies were kept at 23 °C for 3 days or at 29 °C for 2 days before imaging. The *UAS.rho*^*V14*^ and *UAS.rho*^*N19*^ pupae were filmed 1 day after heat-shock.

Pupae carrying clones that expressed various *UAS-*transgenes (*UAS.X*) marked with mCD8-GFP, mCD8-RFP or myr-RFP had the following genotypes:

1) *y w hs.FLP; UAS.mCD8-GFP, tub-FRT-CD2-FRT-Gal4, UAS.FLP/+* or *Sp* or *UAS.X; +/+* or *UAS.X*
2) *y w hs.FLP; UAS.mCD8-RFP, tub-FRT-CD2-FRT-Gal4, UAS.FLP/ubi.DE-cadherin::GFP; MKRS/+*
3) *y w hs.FLP; UAS.myrRFP, UAS.myrRFP, tub-FRT-CD2-FRT-Gal4, UAS.FLP/ ubi.DE-cadherin::GFP, UAS.X; +* or *MKRS/+* or *UAS.X*

Transgenes on either chromosome 2 or 3 were used, with the exception of *UAS.tkv*^*DN*^ where insertions in both chromosomes were used. No differences in phenotype were observed between flies carrying wild-type chromosomes and chromosomes with *Sp* and/or *MRS/MKRS*.

### RNA interference

RNAi lines were obtained from the VDRC (Dietzl et al., 2007) and the BDSC. Various observations made here and by others suggested that the RNAi phenotypes are specific to a knock-down of the targeted gene and also allowed an assessment of the strength of the knock-down:

- *UAS.rho-RNAi*: *UAS.rho*^*N19*^ pupae have a similar phenotype. *UAS.rho-RNAi* has been shown to generate abdominal closure defects when using another driver than in this study (Sekyrova et al., 2010).
- *UAS.ds-RNAi*: The adult cuticle in *UAS.ds-RNAi pupae* shows PCP defects similar to *ds* mutants (data not shown). *UAS.ectoDs* pupae have a similar phenotype.
- *UAS.tkv-RNAi*, *UAS.dpp-RNAi*: *UAS.tkv*^*DN*^ and *UAS.dad* have similar phenotypes. *UAS.dpp-RNAi* was shown to interfere with histoblast development and Dpp is expressed in LECs (Ninov et al., 2010).

### 4D microscopy

For imaging, pupae were staged according to (Bainbridge and Bownes, 1981). A window in the pupal case was made and the pupae were filmed as described in Escudero et al. (2007). The analysis focused on the dorsal side (tergite) of the abdomen. All imaged flies developed into pharate adults and many hatched. *Z*-stacks of around 40 μm with a step size of 2.5 or 3.0 μm were recorded every 150 or 180 s using a Leica SP5 confocal microscope at 23 ± 2 °C. Each genotype was recorded at least three times (Supplementary Table 1). All images and movies shown are projections of *z*-stacks. Figures and movies were made using Adobe Illustrator, Adobe Image Ready, Adobe Photoshop, ImageJ (NIH, Bethesda, USA), Volocity (Improvision), and Quicktime Pro (Apple Inc.).

### Analysis of 4D movies

To analyse cell behaviour in detail, the LECs were tracked manually using the software SIMI Biocell (Schnabel et al., 1997). The 3D coordinates of the nuclei (for H2AvGFP and mCD8GFP) or the centres of the cells (for DE-cadherin::GFP) were saved at least every 30 min.

Trajectory plots were calculated using a programme written in C# using Microsoft Visual Studio 2005 with the Microsoft .NET 2.0 framework (Bischoff and Cseresnyes, 2009). All calculations were performed in two dimensions due to the planar character of the epithelial sheet.

1) Trajectory plots: To display the trajectories of the cells, the coordinates of a cell at a certain time and the coordinates of the same cell 30 min later were connected with a straight ‘beeline’. The colour of these lines represents the velocity of the cell using a banded look-up table. If nuclei were tracked, the trajectories of the cells not only represent the movement of the cell *per se* but also the movement of the nucleus within the cell — for the general direction of the movement, however, this is negligible. In most figures, two migration phases are shown separately — ‘posterior’ and ‘dorsal’. These two phases represent the time intervals in which cells mainly move posteriorly (up to approx. 28 h after puparium formation (APF)) and dorsally (from approx. 28 h APF onwards), respectively.
2) Measurement of the speed of abdominal closure: The average speed of the dorsally expanding histoblast mass was measured from the beginning of dorsal LEC migration to its halt when there were about five rows of LECs left.
3) The relative fluorescence of DE-cadherin::GFP was measured along a line in anterio-posterior (a–p) direction using LAS AF Lite (Leica Microsystems, Mannheim, Germany).
4) The length and width of LECs were measured manually using ImageJ (NIH, Bethesda, USA), at the time when LECs have undergone cell shape changes and started to move (around 26 h APF). The longest line fitting in the apical area of the LECs of segment A2 along their a–p and dorso-ventral (d–v) axes, respectively, was measured.

## Results

### LECs undergo a transition from stationary to migratory behaviour

**Fig. 1:**
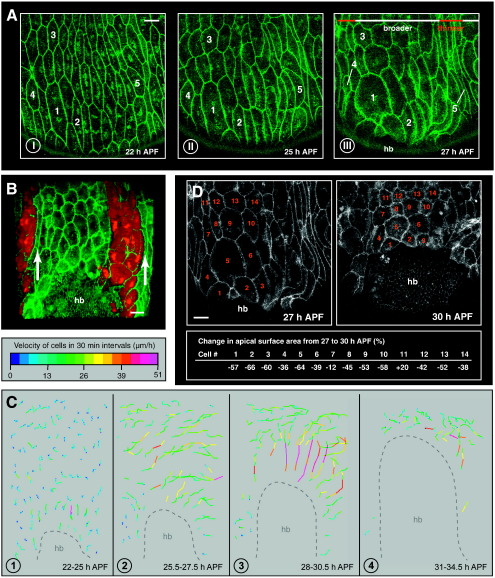
Shape changes and migrations of LECs. In all images, a hemisegment of segment A2 is shown. Anterior is to the left. Cell outlines are marked with DE-cadherin::GFP. APF, after puparium formation. hb, histoblasts. Scale bars, 25 μm. (A) LECs change shape before the onset of migration. These shape changes occur mainly in a–p direction. Most cells in the centre of the segment become wider (*e.g.* #1, 2, 3), whereas those at the segment boundaries become narrower (*e.g.* #4, 5). Before the shape changes, LECs are more or less hexagonally shaped. (I). LECs shortly before (II) and after the onset of migration (III). See Supplementary Movie 2. (B) Spatial pattern of LEC shape changes. The P compartment is labelled by RFP driven by *en.Gal4*. Cells that are positioned close to the segment boundaries are deformed most (arrows). (C) Migration of the LECs, shown in four phases. LEC trajectories are plotted by connecting the coordinates of a cell in 30-minute intervals with a line. The colour represents the velocity of the cells. Over time, the histoblasts replace the LECs. (1) Beginning of histoblast spreading; LECs change shape. Then LECs first move posteriorly (2), then dorsally (3). Note that the dorsal migrations are the fastest. (4) Final apical constriction of LECs before the histoblasts of both hemisegments meets at the midline. (D) While migrating, LECs also constrict apically. The pattern of LEC constriction is shown by displaying the change in surface area of some LECs from 27 to 30 hour APF. Independent of their position in the segment, cells constrict apically, although to different extents. Shape changes and migrations of LECs. In all images, a hemisegment of segment A2 is shown. Anterior is to the left. Cell outlines are marked with DE-cadherin::GFP. APF, after puparium formation. hb, histoblasts. Scale bars, 25 μm. (A) LECs change shape before the onset of migration. These shape changes occur mainly in a–p direction. Most cells in the centre of the segment become wider (*e.g.* #1, 2, 3), whereas those at the segment boundaries become narrower (*e.g.* #4, 5). Before the shape changes, LECs are more or less hexagonally shaped. (I). LECs shortly before (II) and after the onset of migration (III). See Supplementary Movie 2. (B) Spatial pattern of LEC shape changes. The P compartment is labelled by RFP driven by *en.Gal4*. Cells that are positioned close to the segment boundaries are deformed most (arrows). (C) Migration of the LECs, shown in four phases. LEC trajectories are plotted by connecting the coordinates of a cell in 30-minute intervals with a line. The colour represents the velocity of the cells. Over time, the histoblasts replace the LECs. (1) Beginning of histoblast spreading; LECs change shape. Then LECs first move posteriorly (2), then dorsally (3). Note that the dorsal migrations are the fastest. (4) Final apical constriction of LECs before the histoblasts of both hemisegments meet at the midline. (D) While migrating, LECs also constrict apically. The pattern of LEC constriction is shown by displaying the change in surface area of some LECs from 27 to 30 hours APF. Independent of their position in the segment, cells constrict apically, although to different extents.

The LECs are large cells (up to 70 μm in apical diameter), which makes them well suited to study cell behaviour *in vivo*. I analysed LEC behaviour from around 20 hours APF – when the histoblast nests start spreading – until the closure of the epithelium at around 41 hours APF and found that the LECs undergo a transition from stationary to migratory behaviour. This transition is associated with a shape change — the hexagonal LECs become roundish by expanding (spreading) mainly along the a–p axis (Fig. 1A; Supplementary Movie 2). Cell shape changes mainly occur in the centre of the segment and lead to a compaction of those cells that are located close to the segment boundary (Fig. 1B).

Then the cells start to migrate. These migrations are extensive and directed (Fig. 1C; Supplementary Movies 2, 3). Initially, the majority of cells move posteriorly; later the LECs turn and move dorsally towards the midline. The dorsal migrations are faster than the posterior migrations (Fig. 1C). While migrating, the LECs also constrict apically (Fig. 1D). Importantly, the LECs never lose contact with their neighbours and thus remain as a coherent epithelium throughout morphogenesis (Ninov et al., 2010; Supplementary Movie 2).

### LECs migrate using apical crescent-shaped protrusions

**Fig. 2:**
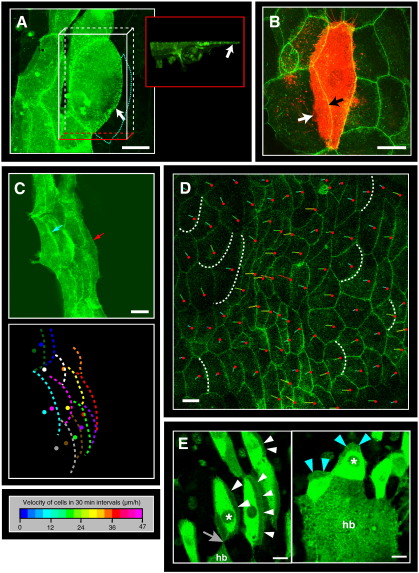
Migrating LECs produce apical lamellipodia-like protrusions that are oriented in the direction of movement. In all images, anterior is to the left. Scale bars, 25 μm. (A–C) All cells are marked with DE-cadherin::GFP. (A) Apical crescent-shaped protrusion of a LEC during posterior migration (arrow). View from above (white box) and from the side (red box). The cell body is marked by overexpressing GFP-actin with *en.Gal4*. A neighbouring cell (highlighted by cyan dotted line) is strongly deformed by the protrusion. (B) Apically, LECs move ‘on top’ of each other. Single-cell clone is shown, marked by mCD8-RFP. Arrows point at the cell–cell boundary in the most apical (black) and the most basal (white) slice of this *z*-projection. The left neighbour is moving ‘on top’ of the RFP-marked cell. (C) During the posterior migration phase, LECs generate protrusions in posterior direction and move partly ‘on top’ of their neighbours. One frame of Supplementary Movie 4 is shown. In the cartoon, the protrusions of the individual LECs are highlighted with coloured hatched lines and the cells' nuclei are marked with a dot of the same colour. Most of the nuclei are positioned beneath a protrusion of a more anteriorly positioned neighbour. All cells generate protrusions in posterior direction so that protrusions overlap in a planar polarised fashion. (D) Merge of a confocal image and a trajectory plot. The plot shows the direction of movement within the next 30 min. The red dots indicate the direction of movement. Most LECs produce crescent-shaped protrusions, which in most cases point in the direction of movement (some protrusions are highlighted with dotted lines). See Supplementary Movie 5. (E) The LECs generate their protrusions in the direction of movement. White arrowheads show posterior, blue arrowheads dorsal protrusions. Two frames taken from Supplementary Movie 6 are shown. Furthermore, the histoblasts appear to be able to repolarise the migrating LECs: The LEC marked with an asterisk is repolarised when approached by the histoblasts (hb). In the first frame, the cell shows a protrusion in posterior direction and is being approached by the histoblasts (grey arrow). In the second frame, it shows a dorsal protrusion. Migrating LECs produce apical lamellipodia-like protrusions that are oriented in the direction of movement. In all images, anterior is to the left. Scale bars, 25 μm. (A–C) All cells are marked with DE-cadherin::GFP. (A) Apical crescent-shaped protrusion of a LEC during posterior migration (arrow). View from above (white box) and from the side (red box). The cell body is marked by overexpressing GFP-actin with *en.Gal4*. A neighbouring cell (highlighted by cyan dotted line) is strongly deformed by the protrusion. (B) Apically, LECs move ‘on top’ of each other. Single-cell clone is shown, marked by mCD8-RFP. Arrows point at the cell–cell boundary in the most apical (black) and the most basal (white) slice of this *z*-projection. The left neighbour is moving ‘on top’ of the RFP-marked cell. (C) During the posterior migration phase, LECs generate protrusions in posterior direction and move partly ‘on top’ of their neighbours. One frame of Supplementary Movie 4 is shown. In the cartoon, the protrusions of the individual LECs are highlighted with coloured hatched lines and the cells' nuclei are marked with a dot of the same colour. Most of the nuclei are positioned beneath a protrusion of a more anteriorly positioned neighbour. All cells generate protrusions in posterior direction so that protrusions overlap in a planar polarised fashion. (D) Merge of a confocal image and a trajectory plot. The plot shows the direction of movement within the next 30 min. The red dots indicate the direction of movement. Most LECs produce crescent-shaped protrusions, which in most cases point in the direction of movement (some protrusions are highlighted with dotted lines). See Supplementary Movie 5. (E) The LECs generate their protrusions in the direction of movement. White arrowheads show posterior, blue arrowheads dorsal protrusions. Two frames taken from Supplementary Movie 6 are shown. Furthermore, the histoblasts appear to be able to repolarise the migrating LECs: The LEC marked with an asterisk is repolarised when approached by the histoblasts (hb). In the first frame, the cell shows a protrusion in posterior direction and is being approached by the histoblasts (grey arrow). In the second frame, it shows a dorsal protrusion.

When they start to migrate, the LECs form a single crescent-shaped protrusion at their apical side (Fig. 2A). These protrusions include the adherens junctions and displace the apical area of neighbouring cells. Hence, cells are partly positioned ‘on top’ of their neighbours, overlapping like roof tiles (Figs. 2B, C). The protrusions are highly dynamic and resemble lamellipodia of migrating cells in culture (Supplementary Movie 4). Most LECs produce these protrusions, and the direction of movement correlates with the orientation of the protrusions (Fig. 2D; Supplementary Movie 5). Posteriorly migrating LECs generate protrusions that point posteriorly (Figs. 2C, E), whereas dorsally migrating LECs show protrusions in dorsal direction (Fig. 2E; Supplementary Movies 2, 3, 6). Overall this suggests that the LECs use these protrusions to move.

Interestingly, LECs that migrate posteriorly can repolarise in dorsal direction (Fig. 2E; Supplementary Movie 6). This repolarisation appears to depend on the vicinity of the histoblasts, suggesting that the histoblasts provide a polarising signal.

### LECs move on their apical surface

**Fig. 3:**
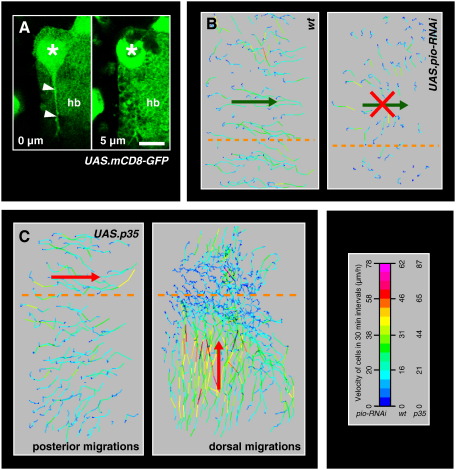
Interfering with apical adhesion and death of the LECs. In all images, anterior is to the left. (A, B) LECs use apical protrusions to move on their apical surface. (A) Retraction fibres are attached apically, above the histoblasts. LECs (asterisks) and histoblasts (hb) are marked with mCD8-GFP. A retraction fibre (arrowheads) can be seen in an apical *z*-plane (left frame; 0 μm) but not in a *z*-plane 5 μm below, where the histoblasts are situated (right frame). Scale bar, 25 μm. (B) Comparison of migration of wild-type LECs and LECs that express *pio-RNAi*. Segment A2 shown. The trajectory plots show the posterior migration phase. *Pio-RNAi* LECs do not migrate posteriorly. (C) Inhibition of LEC death by expression of *UAS.p35*. The trajectory plots of the posterior and dorsal migration phase show that LECs migrate more or less normally. The LECs move posteriorly and dorsally, respectively (indicated by red arrows). Segment A2 shown. The legend shows the look-up table for the velocities of the trajectory plots. Orange dashed lines indicate dorsal midline.

How do the apical protrusions propel the cells? LECs are located between an apical pupal cuticle and a basal lamina, which is degraded before histoblasts start spreading (Ninov et al., 2010). Retraction fibres of the LECs can be observed regularly, which are attached apically above the progressing histoblasts (Fig. 3A). This suggests that the LECs adhere to an apical substrate. An adhesion to the pupal cuticle has been suggested to play a role in histoblast movement, and proteins such as Piopio (Pio) may act in this adhesion (Ninov et al., 2010). Pio is a ZP-domain-containing transmembrane protein involved in the apical adhesion of cells of the pupal wing and embryonic epidermis (Bökel et al., 2005). It is expressed apically in histoblasts and LECs (Ninov et al., 2010). I thus tested whether Pio plays a role in LEC movement. Knock-down of Pio in LECs by RNAi impairs their posterior migration (Fig. 3B). This suggests that apical adhesion mediated by Pio is needed for LEC migration.

Taken together, my results show that LECs migrate using lamellipodia-like apical protrusions, which enable them to move on their apical surface.

### LEC migration is not linked to cell death

What signals regulate LEC migration? LEC migration could be the consequence of the induction of the apoptosis programme. Individual LECs undergo apoptosis throughout morphogenesis, with some LECs already dying during their transition to migratory behaviour (Supplementary Movie 3), but the majority of LECs dying when the histoblast nests expand and the LECs constrict and migrate (Bischoff and Cseresnyes, 2009; Nakajima et al., 2011; Ninov et al., 2007). However, some LECs migrate for up to 9 h before delamination and two reporters for caspase-induced cell death showed that the caspase-pathway in the LECs is only activated approximately 30 min before they die (Nakajima et al., 2011; L.A. Baena-Lopez, R. McGregor, M. Bischoff and J.-P. Vincent, manuscript in preparation). This suggests that LECs migrate before they undergo apoptosis and not as a result of it.

To further test this, LEC death was prevented and it was asked whether the migrations still occurred. P35 as well as Inhibitor-of-Apoptosis-Proteins DIAP1 and DIAP2, which all inhibit the apoptosis cascade at different levels (Hay et al., 1994, 1995), were overexpressed in the LECs. In all three experiments, most LECs did not undergo apoptosis, which lead to a morphogenesis defect as the surviving LECs accumulated at the dorsal midline (Supplementary Figs. 1A–C). This is in accordance with previous reports for the overexpression of P35 (Ninov et al., 2007) and DIAP1 (Sekyrova et al., 2010). However, the LECs migrate normally (Fig. 3C; Supplementary Figs. 1D, E; Supplementary Movie 7), which suggests that LEC migration is a discrete programme that is executed before the LECs die.

### Autocrine Dpp signalling is required for normal migratory behaviour

**Fig. 4:**
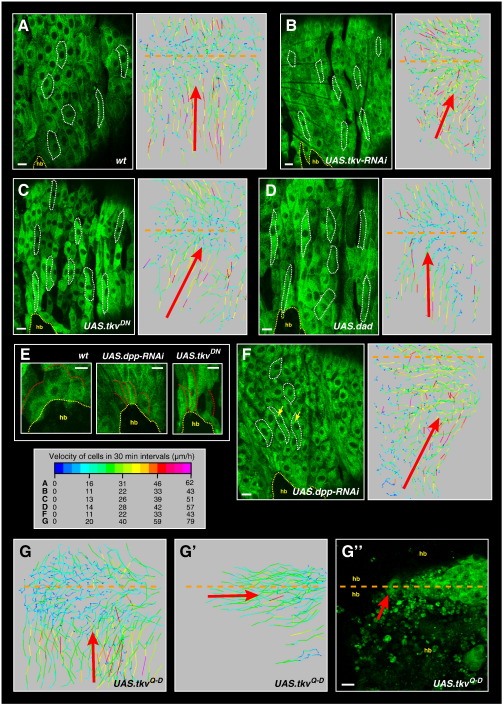
Dpp signalling affects different aspects of LEC motility. Segment A2 and parts of neighbouring segments shown. Some cell outlines are highlighted by white dotted lines. The interface between LECs and histoblasts (hb) is highlighted by yellow dotted lines. Anterior is to the left. LECs are marked with mCD8-GFP. Trajectory plots show dorsal migration phase. Orange dashed line indicates dorsal midline. The legend shows the look-up table for the velocities of the trajectory plots. Scale bars, 25 μm. (A) Wild-type. Many cells have changed shape by getting spreading in a–p direction, which makes them appear only slightly elongated in d–v direction. Cells move straight towards the dorsal midline (red arrow). (B–F) Interfering with Dpp signalling in LECs impairs aspects of their motility. Cells do not change shape along the a–p axis and thus still appear elongated in d–v direction. (B) RNAi knock-down of Tkv. (C) Overexpression of a dominant negative form of Tkv. (D) Overexpression of Dad. In B and C, the dorsal migrations are tilted posteriorly; in D, LECs do not migrate in posterior direction (Supplementary Movie 8) and move straight towards the midline (red arrows). (E) LECs that touch the histoblasts during dorsal migration. Wild-type LECs produce dorsally oriented protrusions, whereas Dpp signalling-deficient cells do not. Protrusions are highlighted by red dotted lines. (F) RNAi knock-down of Dpp in LECs. A few cells do not change shape along the a–p axis and thus still appear elongated in d–v direction (yellow arrows). Dorsal migrations are tilted posteriorly (red arrow). (G) Expression of a constitutively active form of Tkv stimulates migratory behaviour (Supplementary Movie 9). After dorsal migration (G), the LECs continue to migrate posteriorly (G’) instead of ceasing their movement and delaminating. 80% of the recorded pupae show a dorsal closure defect in the adult epithelium due to persisting LECs (*n* = 5). (G”) Confocal image of the posteriorly moving LECs. Anterior to the red arrow, histoblasts from both hemisegments have met at the dorsal midline. Dpp signalling affects different aspects of LEC motility. Segment A2 and parts of neighbouring segments shown. Some cell outlines are highlighted by white dotted lines. The interface between LECs and histoblasts (hb) is highlighted by yellow dotted lines. Anterior is to the left. LECs are marked with mCD8-GFP. Trajectory plots show dorsal migration phase. Orange dashed line indicates dorsal midline. The legend shows the look-up table for the velocities of the trajectory plots. Scale bars, 25 μm. (A) Wild-type. Many cells have changed shape by spreading in a–p direction, which makes them appear only slightly elongated in d–v direction. Cells move straight towards the dorsal midline (red arrow). (B–F) Interfering with Dpp signalling in LECs impairs aspects of their motility. Cells do not change shape along the a–p axis and thus still appear elongated in d–v direction. (B) RNAi knock-down of Tkv. (C) Overexpression of a dominant negative form of Tkv. (D) Overexpression of Dad. In B and C, the dorsal migrations are tilted posteriorly; in D, LECs do not migrate in posterior direction (Supplementary Movie 8) and move straight towards the midline (red arrows). (E) LECs that touch the histoblasts during dorsal migration. Wild-type LECs produce dorsally oriented protrusions, whereas Dpp signalling-deficient cells do not. Protrusions are highlighted by red dotted lines. (F) RNAi knock-down of Dpp in LECs. A few cells do not change shape along the a–p axis and thus still appear elongated in d–v direction (yellow arrows). Dorsal migrations are tilted posteriorly (red arrow). (G) Expression of a constitutively active form of Tkv stimulates migratory behaviour (Supplementary Movie 9). After dorsal migration (G), the LECs continue to migrate posteriorly (G’) instead of ceasing their movement and delaminating. 80% of the recorded pupae show a dorsal closure defect in the adult epithelium due to persisting LECs (*n* = 5). (G”) Confocal image of the posteriorly moving LECs. Anterior to the red arrow, histoblasts from both hemisegments have met at the dorsal midline.

To further study the mechanisms underlying the regulation of LEC migration, I next tested whether Dpp signalling plays a role in LEC migration. In the abdomen, Dpp has been shown to direct histoblast motility (Ninov et al., 2010), and vertebrate Dpp homologues are implicated in stimulating cell migration (de Jesus Perez et al., 2011; Gamell et al., 2008; Kang et al., 2010). Dpp signalling in the LECs was manipulated in three different ways: (i) Knock-down of the Dpp receptor Thickveins (Tkv) by RNAi, (ii) overexpression of a dominant negative form of Tkv (Tkv^DN^ (Haerry et al., 1998)), and (iii) overexpression of Daughters against dpp (Dad) (Figs. 4A–D; Supplementary Table 2, Supplementary Movie 8). Dad is a Dpp target gene that inhibits Dpp signalling (Tsuneizumi et al., 1997).

In all three experiments, LECs show impaired migratory behaviour. In most cases, the dorsal movements are affected — they are tilted posteriorly (Figs. 4B, C), and the LECs lack dorsally directed protrusions (Fig. 4E). In strong cases, the posterior migrations are absent (Supplementary Movie 8; Supplementary Table 2). In this case, also the tilt in the dorsal movements is absent (Fig. 4D), which implies that this tilt might be due to posterior LEC migration — the LECs continue to move posteriorly once the apical constrictions have begun, instead of repolarising dorsally. Additionally, in many cases, the LECs fail to change shape and spread along the a–p axis before they start moving, so that many cells still appear elongated along the d–v axis (Figs. 4B–D; Supplementary Fig. 2; Supplementary Table 2).

Overall, these results suggest that Dpp signalling is required for normal migratory behaviour of the LECs, since interfering with Dpp signalling impairs different aspects of LEC motility. Differences in the strength of the phenotypes could be due to different time points at which the cells ‘run out of’ enough Dpp signalling to obtain or maintain their migratory phenotype.

Interestingly, the knock-down of Dpp only in the LECs leads to a phenotype comparable to a manipulation of Dpp reception (Fig. 4F; Supplementary Table 2). This suggests that the Dpp signal that stimulates LEC migration is produced by the LECs themselves. Such an autocrine Dpp signal has been suggested to prevent the premature death of the LECs (Ninov et al., 2010).

To further test the role of Dpp signalling in LEC migrations, Dpp signalling was stimulated in the LECs by the overexpression of a constitutively active form of Tkv (Tkv^Q-D^ (Nellen et al., 1996)). This delays the death of the LECs (Ninov et al., 2010), which accumulate at the dorsal midline (Fig. 4G; Supplementary Movie 9). Unlike LECs, in which cell death inhibitors are expressed and which stop moving after they have accumulated at the midline (Supplementary Movie 7), the *tkv*^*Q-D*^ LECs continue to move. Thus, besides implicating a role of Dpp signalling in LEC survival, the constitutive activation of Tkv corroborates the finding that Dpp signalling stimulates migratory behaviour.

### The posterior migrations depend on planar cell polarity (PCP) genes

**Fig. 5:**
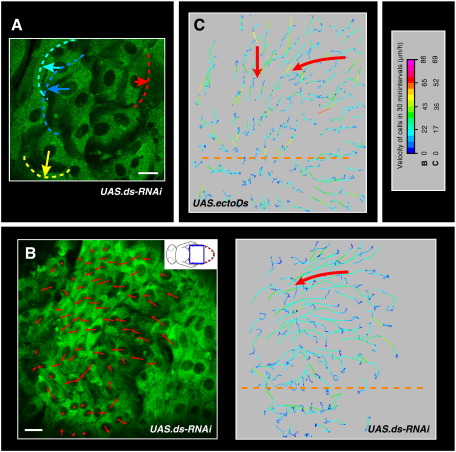
The directionality of posterior LEC migration depends on the PCP gene Ds. In all images, cells are marked with mCD8-GFP. Anterior is to the left. The legend shows the look-up table for the velocities of the trajectory plots in B and C, which show the posterior migration phase. Orange dashed lines indicate dorsal midline. Scale bars, 25 μm. (A, B) Pupae, in which most LECs express *UAS.ds-RNAi*. (A) Part of segment A2 shown. At the beginning of their movement, LECs move haphazardly. The protrusions are highlighted with dotted lines and the direction of movement with arrows. (B) Left panel: Merge of a confocal image and a trajectory plot. The arrows show the direction of movement within the next 30 min after the image was taken. Most LECs move anteriorly, although at the same stage cells move posteriorly in wild-type pupae (Figs. 1C, 3B). Cartoon shows depicted area, consisting of segment A2 and parts of neighbouring segments. Right panel: Trajectory plot. The red arrow indicates direction of movement. (C) Trajectory plot of a pupa, in which most LECs express *UAS.ectoDs*. The LECs move dorsally or anteriorly but not posteriorly (highlighted by red arrows).

LEC migrations are highly directed, but what determines their orientation? For the posterior movements, cells could use information provided by the existing polarity of the larval epithelium. The larval epithelium is polarised in a–p direction, which manifests itself in the directed outgrowth of denticles (Lohs-Schardin et al., 1979). As the atypical cadherin Dachsous (Ds) is involved in larval PCP (Casal et al., 2006; Repiso et al., 2010), I tested whether knock-down of Ds by RNAi in the LECs interferes with the orientation of their apical protrusions and with the direction of their migration. In 93% of the analysed pupae, expression of *ds-RNAi* in the LECs leads to a loss of directionality of the movements (*n* = 14; Figs. 5A, B). Cells start to create protrusions and to move haphazardly in different directions (Fig. 5A), resulting in a net neutralisation of the posterior movements (Supplementary Movie 10). In one pupa, whole regions of the epithelium move anteriorly instead of posteriorly (Fig. 5B). The subsequent dorsal migrations and the apical constrictions of the LECs appear to be unaffected, so that normal abdomen develop (Supplementary Movie 10).

A similar phenotype was obtained by overexpressing the extracellular domain of Ds (*UAS.ectoDs*) in LECs, which has been shown to induce PCP phenotypes in adult and larval epithelia (Casal et al., 2006; Repiso et al., 2010). In all recorded pupae, LEC migration loses directionality and posterior migration is neutralised (*n* = 6; data not shown). In 50% of pupae, whole regions of the epithelium move anteriorly (Fig. 5C).

These results suggest that the LECs use the planar polarity of the epithelium, which depends on Ds, to determine the initial direction of their movement.

### Activation of Rho1 induces apical constriction

As mentioned above, while migrating, the LECs also constrict apically (Supplementary Movie 2; Fig. 1D; (Ninov et al., 2007)). In order to assess how these two behaviours are integrated, I manipulated levels of RhoA (Rho1 in *Drosophila* (Hariharan et al., 1995)) in the LECs. This small Rho GTPase is a regulator of acto-myosin contractility (Etienne-Manneville and Hall, 2002; Ridley and Hall, 1992) and is crucial for apical constriction (Barrett et al., 1997; Sawyer et al., 2010). Rho1 signalling also acts in the delamination of the LECs (Ninov et al., 2007; Sekyrova et al., 2010).

**Fig. 6:**
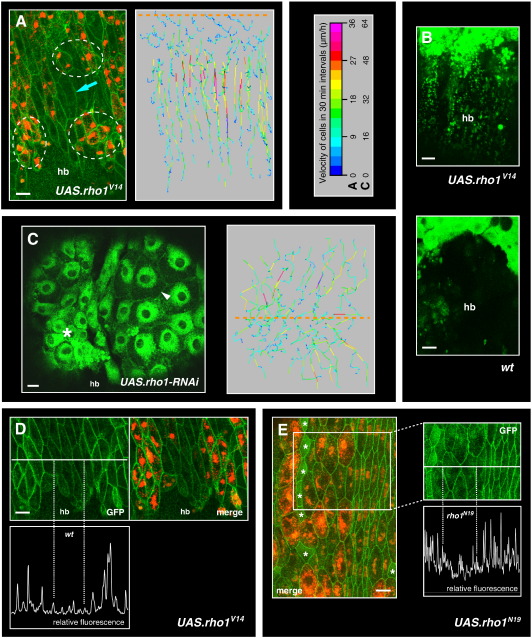
The role of Rho1 in apical constriction and migration of the LECs. In all images, anterior is to the left. The legend shows the look-up table for the velocities of trajectory plots in A and C. Orange dashed lines indicate dorsal midline. hb, histoblasts. Scale bars, 25 μm. (A, B) Expression of a constitutively active form of Rho1 (Rho1^V14^) in LECs. Hemisegments of segment A2. (A) Overexpression of Rho1^V14^ in clones of LECs (RFP-positive) leads to apical constriction (see Supplementary Movie 11). All cells express DE-cadherin::GFP. Many Rho1^V14^ LECs have already begun to constrict apically (white circles), whereas those cells, which do not yet express Rho1^V14^ or have only just started to do so, have not yet constricted. Some Rho1^V14^-negative cells produce crescent-shaped protrusions (*e.g.* blue arrow). The trajectory plot shows that LECs do not move posteriorly but straight towards the midline, at lower speeds than in wild-type (Fig. 1C). (B) Rho1^V14^ expressing LECs marked with mCD8-GFP. LECs leave GFP-positive ‘footprints’ approximately 10 μm apical to the histoblasts (Supplementary Movie 12). (C) Knock-down of Rho1 by RNAi (Supplementary Movie 13). LECs expressing *UAS.rho-RNAi* are marked with mCD8-GFP. The confocal image shows LECs that have started to constrict (asterisk) and others that display extensive spreading with increased apical area (arrowhead). The trajectory plot shows that the directionality of the movement is impaired. (D) The Rho1^V14^ expressing LECs (RFP-positive) have more DE-cadherin::GFP at their adherens junctions than those LECs that have not yet started to express Rho1^V14^ (RFP-negative), although they have the same size. Same pupa as in A. Relative fluorescence has been quantified along the white line. The dotted lines indicate the area of junctions between adjacent wild-type LECs. (E) Overexpression of Rho1^N19^ in clones of LECs that are marked with RFP. All cells are marked with DE-cadherin::GFP. The expression of Rho1^N19^ leads to spreading of cells compared to wild-type cells (white asterisks) and cells that have only just started to express Rho1^N19^ (cells with less intense RFP-staining). The cells with the high RFP expression have lower DE-cadherin::GFP-levels at their adherens junctions, which are highlighted by quantifying the relative fluorescence along the white line. The dotted lines indicate the area of junctions between neighbouring Rho1^N19^ expressing LECs. The role of Rho1 in apical constriction and migration of the LECs. In all images, anterior is to the left. The legend shows the look-up table for the velocities of trajectory plots in A and C. Orange dashed lines indicate dorsal midline. hb, histoblasts. Scale bars, 25 μm. (A, B) Expression of a constitutively active form of Rho1 (Rho1^V14^) in LECs. Hemisegments of segment A2. (A) Overexpression of Rho1^V14^ in clones of LECs (RFP-positive) leads to apical constriction (see Supplementary Movie 11). All cells express DE-cadherin::GFP. Many Rho1^V14^ LECs have already begun to constrict apically (white circles), whereas those cells, which do not yet express Rho1^V14^ or have only just started to do so, have not yet constricted. Some Rho1^V14^-negative cells produce crescent-shaped protrusions (*e.g.* blue arrow). The trajectory plot shows that LECs do not move posteriorly but straight towards the midline, at lower speeds than in wild-type (Fig. 1C). (B) Rho1^V14^ expressing LECs marked with mCD8-GFP. LECs leave GFP-positive ‘footprints’ approximately 10 μm apical to the histoblasts (Supplementary Movie 12). (C) Knock-down of Rho1 by RNAi (Supplementary Movie 13). LECs expressing *UAS.rho-RNAi* are marked with mCD8-GFP. The confocal image shows LECs that have started to constrict (asterisk) and others that display extensive spreading with increased apical area (arrowhead). The trajectory plot shows that the directionality of the movement is impaired. (D) The Rho1^V14^ expressing LECs (RFP-positive) have more DE-cadherin::GFP at their adherens junctions than those LECs that have not yet started to express Rho1^V14^ (RFP-negative), although they have the same size. Same pupa as in A. Relative fluorescence has been quantified along the white line. The dotted lines indicate the area of junctions between adjacent wild-type LECs. (E) Overexpression of Rho1^N19^ in clones of LECs that are marked with RFP. All cells are marked with DE-cadherin::GFP. The expression of Rho1^N19^ leads to spreading of cells compared to wild-type cells (white asterisks) and cells that have only just started to express Rho1^N19^ (cells with less intense RFP-staining). The cells with the high RFP expression have lower DE-cadherin::GFP-levels at their adherens junctions, which is highlighted by quantifying the relative fluorescence along the white line. The dotted lines indicate the area of junctions between neighbouring Rho1^N19^ expressing LECs.

LECs that overexpress a constitutively active form of Rho1 (Rho1^V14^ (Fanto et al., 2000)) constrict throughout the tissue soon after Rho1^V14^ expression starts (Fig. 6A; Supplementary Movies 11, 12). Rho1^V14^ expressing LECs do not change shape (spread) in a–p direction, do not generate apical protrusions and do not migrate posteriorly. This suggests that overactivation of Rho1 drives LECs towards apical constriction. Closure proceeds, although significantly more slowly than in wild-type (23 ± 4 *vs*. 32 ± 5 μm/h; *p* < 0.001, Student's *t*-test). Furthermore, 63% of the pharate flies show defects in abdominal closure (*n* = 9). Since no dorsally directed protrusions can be observed, it is conceivable that closure is occurring solely by apical constriction with no contribution of LEC migration.

In experiments where the membranes of Rho1^V14^ overexpressing cells were marked using *UAS.mCD8-GFP*, GFP-positive ‘footprints’ of the constricting cells can be observed, which remain apical to the histoblasts, possibly at the pupal cuticle (Fig. 6B; Supplementary Movie 12). This suggests that the LECs constrict before they can fully detach from their apical substrate. It is furthermore possible that the overexpression of Rho1^V14^, in addition to driving the LECs to constrict, also stabilises the adhesion of the LECs to their apical substrate, as Rho is required for the formation and maintenance of focal adhesions (Ridley and Hall, 1992).

### Down-regulation of Rho1 leads to cell spreading

Since overexpression of Rho1^V14^ drove LECs to constrict without migration, it seemed possible that knock-down of Rho1 activity by RNAi would impair LEC constriction. However, *rho1-RNAi* LECs do constrict (Supplementary Movie 13), but prior to constriction, they spread extensively, which implies excessive protrusive activity (n = 6; Fig. 6C; Supplementary Movie 13). The directionality of LEC movement, however, is impaired, perhaps because the protrusions are poorly oriented. Overall, 67% of the pharate flies show dorsal closure defects associated with LECs that persist. Overexpression of a dominant negative form of Rho1 (Rho1^N19^ (Strutt et al., 1997)) in the LECs leads to a similar phenotype (Supplementary Fig. 3). Thus, down-regulation of Rho1 in LECs has the opposite effect to its activation, as it stimulates cell spreading.

### Rho1 affects cell–cell adhesion

Besides influencing cell behaviour, the manipulation of Rho1 also appears to affect cell–cell adhesion in the LECs. Levels of junctional DE-cadherin::GFP in LECs that overexpress Rho1^V14^ are higher than in wild-type cells, whereas Rho1^N19^ LECs show lower levels at their adherens junctions than wild-type cells (Figs. 6D, E). Since DE-cadherin::GFP was driven by a ubiquitin promotor (Oda and Tsukita, 2001), these results suggest that Rho1 influences junctional DE-cadherin levels post-transcriptionally. Indeed, Rho1 has been shown to maintain adherens junctions by inhibiting DE-cadherin endocytosis in the *Drosophila* pupal eye (Warner and Longmore, 2009). Furthermore, in pupae in which LECs express Rho1^N19^ marked with *mCD8-GFP*, the larval epithelium tears open (n = 3; Supplementary Fig. 3B; Supplementary Movie 14). Together with the observation that junctional DE-cadherin levels are lower in Rho1^N19^ LECs than in wild-type LECs, this suggests that cell–cell adhesion is decreased between Rho1^N19^ cells. A reduction in DE-cadherin and cell–cell adhesion due to expression of Rho1^N19^ has also been shown for *Drosophila* embryonic epithelial cells (Bloor and Kiehart, 2002). Thus, in addition to the regulation of cytoskeletal changes, Rho1 also appears to regulate the strength of cell–cell adhesion between LECs. A reduction in DE-cadherin levels was also observed at the adherens junctions of LECs in wild-type pupae, as they transit from stationary to migratory behaviour (Supplementary Fig. 4, Supplementary Movie 15).

## Discussion

**Fig. 7:**
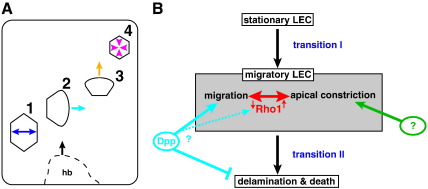
The behaviour of LECs during abdominal closure. (A) Different phases of LEC behaviour. (1) LECs change their shape, mainly along the a–p axis. (2) When the histoblast nests (hb) start to expand dorsally (black arrow), the LECs start to migrate posteriorly (cyan arrow). Posterior migration orientation depends on the planar polarity of the epithelium. (3) LECs repolarise, probably through a signal provided by the histoblasts, and then migrate in dorsal direction (yellow arrow). LECs also constrict apically. (4) The last remaining LECs delaminate (red arrows) and the histoblasts of the two hemisegments meet at the dorsal midline. (B) Model of the regulation of LEC behaviour during abdominal closure. LECs undergo two transitions: (I) LECs transit from stationary to migratory behaviour, showing some aspects of EMT such as the down-regulation of cell–cell adhesion. (II) Only after migration and constriction, the LECs undergo a second transition, which leads to their delamination and death. Rho1 levels can bias cells towards migratory or constrictive behaviour. Dpp signalling stimulates migratory behaviour and promotes cell survival. The observation that both Rho1 and Dpp signalling can affect LEC motility might suggest that at least some aspects of Dpp-stimulated LEC motility might be mediated by Rho1. In wing imaginal discs, Dpp regulates cell shape changes via Rho1 (Widmann and Dahmann, 2009). What signals regulate apical constriction remains unknown.

Here I describe a novel behaviour of the LECs during morphogenesis of the adult abdominal epidermis of *Drosophila* (Fig. 7A). The LECs undergo extensive migrations, generating a single apical lamellipodia-like protrusion (Fig. 2). My results suggest that these protrusions propel the LECs similar to lamellipodia propelling migrating fibroblasts in culture (Abercrombie et al., 1970), yet the LECs do not move on a basal substrate but on their apical surface. Evidence for this comes from a number of experiments: (1) The orientation of the protrusions correlates notably with the direction of movement (Figs. 2C–E; Supplementary Movies 5, 6). (2) LECs that overexpress Rho1^V14^ do not generate protrusions and do not migrate posteriorly (Fig. 6A; Supplementary Movies 11, 12). (3) LECs in which Ds has been knocked-down by RNAi create protrusions in ‘random’ directions and also move in these directions (Fig. 5). (4) LECs in which Pio has been knocked-down by RNAi do not migrate posteriorly, which suggests that the apical protrusions generate traction via adhesion on their apical surface to propel the cells (Fig 3B).

The LECs move as a two-dimensional epithelial sheet rather than individually — they maintain contact with their neighbours, and the integrity of the epithelial sheet is never disrupted (Ninov et al., 2007; Supplementary Movie 2). Thus, the LECs can only move by deforming their neighbours or when their neighbours make space, either by moving or by dying. In such epithelial sheets, cells migrate collectively and the movement of the cells has to be coordinated, *e.g.* by guidance cues or directional mechanical forces (Lecaudey and Gilmour, 2006; Weijer, 2009). A coordination of cell behaviour might also occur between LECs. Some observations, however, suggest that at least with respect to some aspects of their migratory behaviour the LECs behave ‘individually’. Supplementary Movie 6 illustrates that, while some LECs move posteriorly, others repolarise and move dorsally. This repolarisation happens in individual cells rather than in a group. Furthermore, if planar polarity of the tissue is impaired, neighbouring cells generate protrusions and start to move in different directions (Fig. 5A). This suggests that at least initially neighbours do not coordinate the direction of their migration with each other.

### LECs transit from stationary to migratory behaviour

Throughout larval life, the LECs are stationary epithelial cells that secrete cuticle. However this study shows that, during metamorphosis, they become mobile, which involves a transition from stationary to migratory behaviour. This transition is accompanied by a reduction in DE-cadherin levels at the adherens junctions of the LECs (Supplementary Fig. 4; Supplementary Movie 15). These features are characteristic of EMTs (Baum et al., 2008; Huber et al., 2005; Thiery et al., 2009). The LECs' transition is, however, only partial as they do not lose adhesion to their neighbours. This maintenance of cell–cell adhesion might be important to maintain tissue integrity and to avoid the tearing of the tissue, as it can *e.g.* be observed in pupae in which the LECs overexpress Rho1^N19^ (Supplementary Fig. 3B; Movie 14). Yet a reduction in DE-cadherin levels might increase the LECs' ability to move relative to each other.

### The regulation of LEC migration

LEC migrations take place throughout morphogenesis of the adult abdominal epidermis — from the beginning of histoblast spreading, when LECs start to move posteriorly, until the actual closure event, when LECs move dorsally and constrict (Figs. 1D, 7A). So cell motility not only has to be controlled *per se*, but also the directionality of the movement has to be regulated and migratory behaviour has to be coordinated with constrictive behaviour.

#### a) Autocrine Dpp signalling plays a role in LEC motility and survival

My results suggest that autocrine Dpp signalling is required for normal LEC motility. However, unlike other bone morphogenetic proteins (BMPs) that were implicated in inducing EMT (Kang et al., 2010; Yan et al., 2010), Dpp appears not to regulate the transition from stationary to migratory behaviour *per se*. Instead, it appears to stimulate and maintain migratory behaviour, as shown in smooth muscle, epithelial, cancer and stem cells (de Jesus Perez et al., 2011; Eger et al., 2004; Gamell et al., 2008; Kang et al., 2010). As Dpp signalling also directs motility of the histoblasts (Ninov et al., 2010), Dpp affects migratory behaviour of both larval and adult cells, which suggests a general role of Dpp in stimulating motility of different cell types. Moreover, Dpp signalling is involved in LEC delamination — constitutive activation of Dpp signalling delays LEC delamination (Ninov et al., 2010) and leads to LEC survival (Fig. 4G). Dpp might thus act as a survival factor (Adachi-Yamada and O'Connor, 2004; Ninov et al., 2010) or affect the organisation of the cytoskeleton and hence promote cell survival indirectly (Gibson and Perrimon, 2005; Shen and Dahmann, 2005) (Fig. 7B).

#### b) Posterior LEC migration is dependent on PCP

My results show that the LECs use the existing planar polarity of the epithelium, which is mediated by Ds, to determine the initial direction of their migration (Fig. 5; Supplementary Movie 10). To my knowledge, this is the first evidence that the Ds-system, which is one of the two molecular systems that direct PCP (Casal et al., 2006), is involved in directed cell migrations. PCP mediated by non-canonical Wnt signalling, on the other hand, has been shown to play a role in directed cell migration in vertebrates (Heisenberg et al., 2000; Keller, 2002; Matthews et al., 2008).

#### c) Coordination of LEC behaviour by Rho signalling

How is cell motility implemented on the level of individual cells — particularly in the case of the LECs, which also undergo apical constriction during their migration? My results show that the activation of the small GTPase Rho1 inhibits LEC migration and drives LECs to constrict apically (Figs. 6A,B; Supplementary Movies 11, 12); Rho1 knock-down, on the other hand, leads to cell spreading and increased protrusive behaviour (Fig. 6C; Supplementary Movie 13). Thus, Rho1 activity can coordinate two different behaviours by biasing LECs towards either migratory or constrictive behaviour (Fig. 7B). Thus, LECs ‘integrate’ two previously described aspects of Rho function: (1) Rho regulates actin-myosin contractility in epithelial cells, which drives cell shape change (often by apical constriction) (Azevedo et al., 2011; Barrett et al., 1997; Kinoshita et al., 2008; Nikolaidou and Barrett, 2004; Widmann and Dahmann, 2009). (2) Repression of Rho activity stimulates membrane protrusion and inhibits actin-myosin contractility, thereby promoting cell spreading (Huveneers and Danen, 2009; Jackson et al., 2011).

A further role of Rho is to regulate cell–cell adhesion in epithelial cells (Bloor and Kiehart, 2002; Braga et al., 1997; Warner and Longmore, 2009), which also appears to be the case in the LECs; DE-cadherin levels at the adherens junctions correlate with the manipulation of Rho1 activity (Figs. 6D, E). Furthermore, overexpression of Rho1^N19^ in LECs leads to a tearing of the epithelium (Supplementary Fig. 3B; Movie 14). This might be a consequence of the observed decrease of DE-cadherin levels at the adherens junctions of Rho1^N19^ LECs (Fig. 6E).

Thus, this study suggests that Rho1 coordinates both cytoskeletal and adhesive aspects of migratory and constrictive cell behaviour. Such coordination is important, since it is conceivable that migrating cells need to decrease adhesion to move more freely, whereas constricting cells need to increase adhesion to withstand occurring tensions.

### LEC migrations contribute to abdominal closure

What is the role of the migrations in abdominal closure? Expression of Rho1^V14^ in the LECs suggests that constrictions alone can lead to a (passive) movement of LECs towards the dorsal midline — without any LEC migration. However, abdominal closure is less successful in these pupae — it proceeds more slowly and in 63% of the pupae, closure fails. This indicates that dorsal LEC migrations are required for normal closure. Perhaps the migrations create dorsally directed forces, which – together with apical constriction – facilitate closure. These forces could either pull the histoblasts dorsally or create space for the expanding histoblasts by ‘running away’ from them. Manipulating LEC migration by interfering with PCP and Dpp signalling furthermore shows that LEC migration gives closure directionality. Besides apical constriction and migration of the LECs, the active movement of the histoblasts might also contribute to closure (Bischoff and Cseresnyes, 2009; Ninov et al., 2010).

The role of the posteriorly directed migrations, however, remains mysterious. But it is conspicuous that the compartment and segment boundaries in the early pupa are bent anteriorly (Supplementary Fig. 5). Furthermore, the posterior migrations are most extensive around the dorsal midline. A possible role for the posterior migrations could therefore be the alignment of LECs along the d–v axis and thus the straightening of the compartment and segment boundaries. The cell shape changes that occur when cells become mobile might also contribute here. This straightening might aid the generation of forces along the d–v axis needed for normal closure. Against this hypothesis might argue that, in ds-RNAi experiments where an overall posterior movement of the LECs is largely absent, abdominal closure still takes place (Supplementary Movie 10). However, it is possible that apical constriction and dorsal migration of the LECs, which both occur in the ds-RNAi experiments, could be able to generate the forces needed for closure, although cells are not aligned normally. Moreover, some alignment might still occur by the initial cell shape changes, which are also present in the ds-RNAi experiments.

### Conclusion

The discovery of LEC migrations shows that LECs are not merely removed by the histoblasts, as previously proposed (Ninov et al., 2010), but play a more active role in morphogenesis. Furthermore, it illustrates that, although the big polyploid LECs differ notably from the small diploid histoblasts (not least in their behaviour — the LECs die, whereas the histoblasts divide), both cell types show motile behaviour, which in both cases depends on Dpp signalling (this study; Ninov et al., 2010).

The findings of this study also demonstrate that different epithelial closure events rely on different mechanisms — in dorsal closure of the *Drosophila* embryo, for example, the amnioserosa cells constrict but do not migrate (Fernandez et al., 2007; Kiehart et al., 2000). Thus, the study of adult abdominal closure adds new aspects to how epithelial sheets move, which is not only important for the understanding of morphogenesis but also of wound healing (Martin and Parkhurst, 2004). The *in vivo* analysis of LEC migration presented here provides a detailed basis for further investigation of cell migration and EMT. Both are important mechanisms that underlie tumour progression, during which cells become mobile and invade surrounding tissue (Thiery, 2002).

The following are the supplementary materials related to this article.

Supplementary materials.

Supplementary Movie 1 Wild-type development of segment A2. A Histone::GFP marker marks all nuclei. The histoblasts (small nuclei) move towards the midline and replace the LECs (large nuclei). One row of LECs that delaminate later separates the histoblasts of neighbouring segments laterally from each other. Image area is 275 by 514 μm. See Fig. 1 C for trajectory plot.

Supplementary Movie 2 Cell shape changes and migration of the LECs. The development of a hemisegment of segment A2 is shown. The dorsal midline is located outside the top of the image. DE-cadherin::GFP labels all membranes. The small histoblasts replace the big LECs. Before the histoblasts move into the image from the bottom and the LECs start to move in posterior direction, the LECs change their shape. This cell shape change mainly occurs along the a–p axis (green arrow). During posterior migration, cells display crescent-shaped protrusions, which point posteriorly (white arrows). During dorsal migration, the protrusions point in dorsal direction (blue arrows). During the whole process, LECs constrict apically, delaminate and die (red arrow). However, constriction is most extensive while LECs are moving dorsally. Image area is 244 μm^2^. Frames from this movie shown in Figs. 1A, D.

Supplementary Movie 3 Wild-type development. LECs are marked with mCD8-GFP. When LECs undergo the transition from stationary to migratory behaviour, they change shape mainly along the anterior–posterior axis (green arrow). Then cells migrate posteriorly and subsequently dorsally, displaying apical lamellipodia-like protrusions in posterior (white arrows) and dorsal (red arrows) direction, respectively. Note that the cells do not lose contact (see Supplementary Movie 2) — the areas that appear darker only do so because the spreading cells get thinner. Some LECs die before migrations begin. Segment A2 and parts of the neighbouring segments shown. The right hemisegment is moving out of focus in the course of the movie. Image area is 304 μm^2^. hb, histoblasts, which are GFP-negative.
Wild-type development. LECs are marked with mCD8-GFP. When LECs undergo the transition from stationary to migratory behaviour, they change shape mainly along the anterior–posterior axis (green arrow). Then cells migrate posteriorly and subsequently dorsally, displaying apical lamellipodia-like protrusions in posterior (white arrows) and dorsal (red arrows) direction, respectively. Note that the cells do not lose contact (see Supplementary Movie 2) — the areas that appear darker only do so because the spreading cells get thinner. Some LECs die before migrations begin. Segment A2 and parts of the neighbouring segments shown. The right hemisegment is moving out of focus in the course of the movie. Image area is 304 μm2. hb, histoblasts, which are GFP-negative.

Supplementary Movie 4 Posterior migration of the LECs. LECs of the P compartment are labelled with *UAS.gma* (Bloor and Kiehart, 2001) driven by *hh.Gal4*. GMA is an actin-binding fragment of moesin fused with GFP, which labels the actin cytoskeleton. LECs move posteriorly, displaying crescent-shaped lamellipodia-like protrusions (blue arrows). Note that all protrusions point posteriorly and that most protrusions are positioned ‘on top’ of a neighbouring, more posterior cell (see Fig. 2 C). At the posterior boundary of the segment, the protrusions can be seen extending towards the unlabelled neighbours (white arrows). Some LECs produce retraction fibres at their back (green arrows). Some cells delaminate (red arrow). Image area is 206 μm^2^. A frame taken from this movie shown in Fig. 2 C.

Supplementary Movie 5 LECs generate protrusions in the direction of movement. In many cases, the protrusions of the LECs point in the direction of movement. At later stages, cells stop migrating and merely constrict apically. The paths the LECs move in 30 min intervals are merged with confocal images. Line colour indicates the speed of the LECs as shown in Fig. 2. All cells are marked with DE-cadherin::GFP. Image area is 356 μm^2^. A frame taken from this movie shown in Fig. 2D.

Supplementary Movie 6 LECs repolarise when approached by the histoblasts. mCD8-GFP marks membranes of clones of LECs in a hemisegment of segment A2. At the beginning of the movie, the histoblasts move into the image from the bottom (hb). Please focus on the LEC marked with an asterisk. It begins to produce a lamellipodia-like protrusion in posterior direction (cyan arrows), but when approached by the histoblasts (white arrow), it repolarises in dorsal direction. Blue arrows point at a posteriorly directed protrusion of another LEC. When migrating dorsally, the LECs generate dorsally directed protrusions (red arrows). Note that cells constrict while they are moving dorsally. Image area is 237 by 348 μm. Frames taken from this movie shown in Fig. 2E.

Supplementary Movie 7 LECs overexpressing DIAP2 migrate normally. LECs that overexpress *UAS.DIAP2* are marked with mCD8-GFP. Segments A1 to A3 shown. LECs move posteriorly and then dorsally, although persisting LECs seem to limit the space for other cells to move, leading to ‘traffic jams’. Since cells do not delaminate, many LECs remain at the dorsal midline, resulting in a dorsal closure defect. Image area is 367 μm^2^. A single frame from this movie and trajectory plot shown in Supplementary Figs. 1B, E.

Supplementary Movie 8 Overexpression of Dad impairs cell motility. LECs that express *UAS.dad* are marked with mCD8-GFP. Cells do not spread in a–p direction and appear elongated in d–v direction (white bars). Posterior migration is virtually absent. Cells close to the histoblasts become round and drift dorsally (white arrows). Segments A2 and A3 shown. Image area is 283 by 395 μm. A single frame taken from this movie and trajectory plot shown in Fig. 4D.

Supplementary Movie 9 Overexpression of a constitutively active form of Tkv in LECs stimulates their motility. LECs that express *UAS.tkv^Q-D^* are marked with mCD8-GFP. Cells do not die and accumulate at the dorsal midline. Furthermore, cells do not stop moving but continue to migrate posteriorly. Segments A2 and A3 shown. Image area is 303 μm^2^. Frames taken from this movie and trajectory plot shown in Fig. 4G.

Supplementary Movie 10 ds-RNAi in the LECs interferes with their posterior migration. LECs that express *UAS.ds-RNAi* are marked with mCD8-GFP. In pupae, in which most LECs express *UAS.ds-RNAi*, the net posterior movement of the LECs is neutralised because of the lack of directed posterior movement of individual LECs. Two lines along the segment boundaries help to appreciate this lack of posterior movement. Note that the dorsal migrations appear unaffected — cells move straight towards the midline. Segment A2 shown. Image area is 218 by 262 μm.

Supplementary Movie 11 Overexpression of a constitutively active form of Rho1 drives LECs to constrict without posterior migration. One hemisegment of segment A2 shown. Clones of LECs overexpressing *UAS.rho^V14^* are marked with RFP. All cells express DE-cadherin::GFP. Left: GFP-channel; right: merge of GFP- and RFPchannels. LECs that express *UAS.rho^V14^* from the start of the movie constrict without any cell shape change and protrusive activity. LECs that begin to express RFP (and Rho^V14^) later in the movie start to constrict soon after the RFP expression comes up. Some LECs that start RFP expression late generate protrusions before (white arrow). Overall, no posterior migration can be observed, and the dorsal movement is likely to be solely due to constriction of the LECs. Note that those LECs that express *UAS.rho^V14^* from the start show higher DE-cadherin::GFP levels at their junctions compared to the other LECs, even if they have the same size. LECs that start to express RFP (and thus Rho^V14^) at a later stage increase fluorescence at their adherens junctions during the course of the movie. Image area is 203 by 337 μm. Frames from this movie and trajectory plot shown in Figs. 6A, D.

Supplementary Movie 12 Overexpression of a constitutively active form of Rho1 drives LECs towards apical constriction. LECs overexpressing *UAS.rho^V14^* are marked with mCD8-GFP. *UAS.rho^V14^* LECs constrict without showing migratory behaviour — they do not move posteriorly and immediately constrict all over the tissue without cell shape change (spreading) along the a–p axis. Interestingly, also the single row of LECs that persists at the segment boundary in wild-type pupae (see Supplementary Movie 1) is constricting and does hence not remain. LECs leave GFP-positive ‘footprints’ (red arrows), which are positioned approximately 10 μm apical to the histoblasts (a few histoblasts are also GFP-positive (white arrows)). This suggests that cells that are driven to constrict do not properly detach from their apical substrate. Interestingly, the constriction leads to an accumulation of cells at the dorsal midline, and only then extensive cell death occurs (GFP-positive fragments of dead cells can be seen). This suggests that Rho^V14^ drives cells to constrict but does not increase the rate of cell death. Segments A2 and A3 shown. Image area is 338 by 472 μm. A single frame taken from this movie shown in Fig. 6B.
Overexpression of a constitutively active form of Rho1 drives LECs towards apical constriction. LECs overexpressing *UAS.rho^V14^* are marked with mCD8-GFP. *UAS.rho^V14^* LECs constrict without showing migratory behaviour — they do not move posteriorly and immediately constrict all over the tissue without cell shape change (spreading) along the a–p axis. Interestingly, also the single row of LECs that persists at the segment boundary in wild-type pupae (see Supplementary Movie 1) is constricting and does hence not remain. LECs leave GFP-positive ‘footprints’ (red arrows), which are positioned approximately 10 μm apical to the histoblasts (a few histoblasts are also GFP-positive (white arrows)). This suggests that cells that are driven to constrict do not properly detach from their apical substrate. Interestingly, the constriction leads to an accumulation of cells at the dorsal midline, and only then extensive cell death occurs (GFP-positive fragments of dead cells can be seen). This suggests that Rho^V14^ drives cells to constrict but does not increase the rate of cell death. Segments A2 and A3 shown. Image area is 338 by 472 μm. A single frame taken from this movie shown in Fig. 6B.

Supplementary Movie 13 Knock-down of Rho1 by RNAi leads to a spreading of the LECs. LECs overexpressing *UAS.rho1-RNAi* are marked with mCD8-GFP. *UAS.rho1-RNAi* LECs do constrict, but prior to constriction they display extensive spreading with increased apical area (one cell is highlighted with white arrows). Segments A1 to A3 shown. Segment A1 and a part of A2 move out of focus during the course of the movie. Image area is 376 μm^2^. A single frame taken from this movie shown in Fig. 6C.

Supplementary Movie 14 Rho1^N19^ overexpression in LECs eventually leads to a tearing of the epithelium, which may be caused by a reduction in cell–cell adhesion. The epithelium tears open (orange arrows in first frame) and the underlying fat body tissue becomes visible. During the course of the movie, two more areas tear open (orange arrows and dotted lines). The LECs that overexpress Rho1N19 are marked with mCD8-GFP. See also Supplementary Fig. 3B.
Rho1^N19^ overexpression in LECs eventually leads to a tearing of the epithelium, which may be caused by a reduction in cell–cell adhesion. The epithelium tears open (orange arrows in first frame) and the underlying fat body tissue becomes visible. During the course of the movie, two more areas tear open (orange arrows and dotted lines). The LECs that overexpress Rho1^N19^ are marked with mCD8-GFP. See also Supplementary Fig. 3B.

Supplementary Movie 15 The DE-cadherin-GFP signal at the adherens junctions of wild-type LECs gets weaker during the transition from stationary to migratory behaviour. For this movie, Supplementary Movie 2 has been coloured using the ‘fire’ look-up table (ImageJ, Bethesda, USA) to highlight the change in GFP-signal intensity. 150 min into the movie, three LECs are highlighted in the left box, while the right box shows the same three LECs at the start of the movie, which illustrates the decrease in fluorescence at the adherens junctions. The reduction in fluorescence intensity appears not to be due to photo-bleaching, as it is not linear throughout the course of the movie. The development of a hemisegment of segment A2 is shown. The dorsal midline is located just outside the top of the image. DE-cadherin::GFP labels all membranes. Image area is 244 μm^2^.
The DE-cadherin-GFP signal at the adherens junctions of wild-type LECs gets weaker during the transition from stationary to migratory behaviour. For this movie, Supplementary Movie 2 has been coloured using the ‘fire’ look-up table (ImageJ, Bethesda, USA) to highlight the change in GFP-signal intensity. 150 min into the movie, three LECs are highlighted in the left box, while the right box shows the same three LECs at the start of the movie, which illustrates the decrease in fluorescence at the adherens junctions. The reduction in fluorescence intensity appears not to be due to photo-bleaching, as it is not linear throughout the course of the movie. The development of a hemisegment of segment A2 is shown. The dorsal midline is located just outside the top of the image. DE-cadherin::GFP labels all membranes. Image area is 244 μm^2^.
