# Supplementary material for: Lamellipodia-based migrations of larval epithelial cells are required for normal closure of the adult epidermis of Drosophila
Source: Dev Biol. 2012 Mar 1;363-135(1):179–90. doi: 10.1016/j.ydbio.2011.12.033 (PMC3314956; doi:10.1016/j.ydbio.2011.12.033)
Supplement: Supplementary materials. [file mmc1.pdf]

Supplementary Material

Supplementary Fig. 1. Blocking cell death leads to a dorsal closure defect but not to a migration defect. (A-C) LECs, marked with mCD8-GFP, remain in the epithelium due to overexpression of apoptosis inhibitors DIAP1 (A), DIAP2 (B) and P35 (C). Histoblasts are GFP-negative. See also Supplementary Movie 7. (D, E) Trajectory plots of the posterior and dorsal migration phases are depicted for the pupae shown in A and B. Orange dashed lines indicate dorsal midline. Anterior is to the left. Scale bars, 25 μm. The LECs move posteriorly and dorsally, respectively (indicated by red arrows). The legend shows the look-up table for the velocities of the trajectory plots. See Fig. 3C for the trajectory plot of the pupa shown in C.

Supplementary Fig. 2. LECs with impaired Dpp signalling (Dpp$^-$) are narrower than control LECs at the time when LECs in wild-type pupae have changed shape along the a-p axis and start to migrate. (A) Box plot of LEC width (along the a-p axis) in μm. Control LECs that express mCD8-GFP are significantly wider than LECs in all Dpp$^-$ experiments ($p<0.001$, Student's *t*-test). (B) Box plot of LEC length (along the d-v axis) in μm. Control LECs that express mCD8-GFP are not significantly longer than LECs in most of the Dpp$^-$ experiments. Only in the dpp-RNAi experiment LECs are significantly shorter ($p<0.001$; Student's *t*-test). (C) For each individual cell, width is plotted against length. On average, control LECs (red dotted line) are 27% wider than Dpp$^-$ LECs (blue dotted line). 83% of the control cells are wider than the average of the Dpp$^-$ cells, whereas only 46% of the Dpp$^-$ cells are wider than their own average (blue arrow). On the other hand, 49% of the control cells are wider than their own average, whereas only 10% of the Dpp$^-$ LECs are wider than the average of the control LECs (red arrow).

Supplementary Fig. 3. Overexpression of a dominant negative form of Rho1 (Rho1^{N19}) in the LECs. Anterior is to the left. Scale bar, 25 μm. LECs that overexpress Rho1^{N19} are marked with mCD8-GFP. (A) LECs show increased spreading similar to Rho1-RNAi experiments (Fig. 6C). It is difficult to identify cell boundaries, but the spreading of the cells becomes obvious from the GFP-negative areas around the nuclei (asterisks), which are due to the thinness of the cells. (B) Rho1^{N19} overexpression in LECs eventually leads to a tear in the epithelium, which might be caused by reduced cell-cell adhesion. Eventually, the epithelium tears open (red arrows) and the underlying fat body tissue becomes visible (asterisk). The orange dotted line highlights the border between LEC and fat body tissue. See also Supplementary Movie 14.

Supplementary Fig. 4. DE-cadherin::GFP levels at the adherens junctions of wild-type LECs decrease during the transition from stationary to migratory mode. Pupa expresses *ubi.DE-cadherin::GFP* in all cells. Projection of two identical *z*-stacks that were taken in a five-hour interval (approx. 18 to 23 h APF) with the same microscope settings, to compare the intensity of the fluorescence signal. No further scanning took place between these two image acquisitions and laser exposition time was kept to a minimum to avoid photo-bleaching. The 'fire' look-up table (ImageJ, Bethesda, NIH) was applied to the images to highlight the differences in fluorescence intensity between the two stages. The relative fluorescence was quantified along three lines in a-p direction. Since the cells move, these lines have been shifted along the d-v axis to cross the same cells at both time points. Most membranes decrease in relative fluorescence over the five-hour interval. Anterior is to the left. Scale bar, 25 μm. See also Supplementary Movie 15.

Supplementary Fig. 5. Posterior LEC migration straightens the compartment and segment boundaries, which are initially bent anteriorly. In all images anterior is to the left. Orange dashed lines indicate dorsal midline. (A) *en.Gal4* driving *UAS.RFP* in the P compartment shows that the compartment and segment boundaries (white dashed lines) are bent anteriorly. All cells are marked with H2AvGFP. Segment A2 and A3 indicated by white lines. (B) Trajectory plot of wild-type development (same pupa as in Fig. 1C). Most of the posterior migrations occur around the dorsal midline (bracket). (C) *en.Gal4* driving *UAS.GFP-actin* in the P compartment shows that after posterior migration the boundaries (white dashed lines) are straightened.

Supplementary Table 1. Numbers of pupae recorded for each presented experiment.

Supplementary Table 2. Manipulation of Dpp signalling affects different aspects of cell motility. Percentage of pupae that display the three types of cell motility defects in Dpp signalling-manipulated LECs: (1) Cells show no posterior migration. (2) Cells do not become wider along the a-p axis before they start to move (see Supplementary Fig. 2 for a quantification of LEC shapes). (3) Dorsal migrations are tilted posteriorly. The analyses focused on the region of the segment where wild-type cells show the most extensive cell shape changes before they start to move. Overall, *UAS.dpp-RNAi* LECs show the weakest phenotype. This could be due to an only partial knock-down of the protein by a weak RNAi effect. The co-expression of DCR2 (Dietzl et al., 2007) does not enhance the *UAS.tkv-RNAi* phenotype.

Supplementary Movies

To notice all the details in the complex movies please use the arrow keys to play the movies to your liking in Quicktime. In all movies, anterior is to the left (the coordinates are shown at the start of the movies). If in field of view, the dorsal midline is indicated by an orange line. Approximate developmental time in hours APF is shown (hrs : min).

Supplementary Movie 1. Wild-type development of segment A2. A Histone::GFP marker marks all nuclei. The histoblasts (small nuclei) move towards the midline and replace the LECs (large nuclei). One row of LECs that delaminate later separates the histoblasts of neighbouring segments laterally from each other. Image area is 275 by 514 μm. See Fig. 1C for trajectory plot.

Supplementary Movie 2. Cell shape changes and migration of the LECs. The development of a hemisegment of segment A2 is shown. The dorsal midline is located outside the top of the image. DE-cadherin::GFP labels all membranes. The small histoblasts replace the big LECs. Before the histoblasts move into the image from the bottom and the LECs start to move in posterior direction, the LECs change their shape. This cell shape change mainly occurs along the a-p axis (green arrow). During posterior migration, cells display crescent-shaped protrusions, which point posteriorly (white arrows). During dorsal migration, the protrusions point in dorsal direction (blue arrows). During the whole process, LECs constrict apically, delaminate and die (red arrow). However, constriction is most extensive while LECs are moving dorsally. Image area is 244 μm^2. Frames from this movie shown in Fig. 1A, D.

Supplementary Movie 3. Wild-type development. LECs are marked with mCD8-GFP. When LECs undergo the transition from stationary to migratory behaviour, they change shape mainly along the anterior-posterior axis (green arrow). Then cells migrate posteriorly and subsequently dorsally, displaying apical lamellipodia-like protrusions in posterior (white arrows) and dorsal (red arrows) direction, respectively. Note that the cells do not lose contact (see Supplementary Movie 2) – the areas that appear darker only do so because the spreading cells get thinner. Some LECs die before migrations begin. Segment A2 and parts of the neighbouring segments shown. The right hemisegment is moving out of focus in the course of the movie. Image area is 304 μm^2. hb, histoblasts, which are GFP-negative.

Supplementary Movie 4. Posterior migration of the LECs. LECs of the P compartment are labelled with *UAS.gma* (Bloor and Kiehart, 2001) driven by *hh.Gal4*. GMA is an actin-binding fragment of moesin fused with GFP, which labels the actin cytoskeleton. LECs move posteriorly, displaying crescent-shaped lamellipodia-like protrusions (blue arrows). Note that all protrusions point posteriorly and that most protrusions are positioned 'on top' of a neighbouring, more posterior cell (see Fig. 2C). At the posterior boundary of the segment, the protrusions can be seen extending towards the unlabelled neighbours (white arrows). Some LECs produce retraction fibres at their back (green arrows). Some cells delaminate (red arrow). Image area is 206 μm^2. A frame taken from this movie shown in Fig. 2C.

Supplementary Movie 5. LECs generate protrusions in the direction of movement. In many cases, the protrusions of the LECs point in the direction of movement. At later stages, cells stop migrating and merely constrict apically. The paths the LECs move in

30 min intervals are merged with confocal images. Line colour indicates the speed of the LECs as shown in Fig. 2. All cells are marked with DE-cadherin::GFP. Image area is 356 μm^2. A frame taken from this movie shown in Fig. 2D.

Supplementary Movie 6. LECs repolarise when approached by the histoblasts. mCD8-GFP marks membranes of clones of LECs in a hemisegment of segment A2. At the beginning of the movie, the histoblasts move into the image from the bottom (hb). Please focus on the LEC marked with an asterisk. It begins to produce a lamellipodia-like protrusion in posterior direction (cyan arrows), but when approached by the histoblasts (white arrow), it repolarises in dorsal direction. Blue arrows point at a posteriorly directed protrusion of another LEC. When migrating dorsally, the LECs generate dorsally directed protrusions (red arrows). Note that cells constrict while they are moving dorsally. Image area is 237 by 348 μm. Frames taken from this movie shown in Fig. 2E.

Supplementary Movie 7. LECs overexpressing DIAP2 migrate normally. LECs that overexpress *UAS.DIAP2* are marked with mCD8-GFP. Segments A1 to A3 shown. LECs move posteriorly and then dorsally, although persisting LECs seem to limit the space for other cells to move, leading to ‘traffic jams‘. Since cells do not delaminate, many LECs remain at the dorsal midline, resulting in a dorsal closure defect. Image area is 367 μm^2. A single frame from this movie and trajectory plot shown in Supplementary Fig. 1B, E.

Supplementary Movie 8. Overexpression of Dad impairs cell motility. LECs that express *UAS.dad* are marked with mCD8-GFP. Cells do not spread in a-p direction

and appear elongated in d-v direction (white bars). Posterior migration is virtually absent. Cells close to the histoblasts become round and drift dorsally (white arrows). Segments A2 and A3 shown. Image area is 283 by 395 μm. A single frame taken from this movie and trajectory plot shown in Fig. 4D.

Supplementary Movie 9. Overexpression of a constitutively active form of Tkv in LECs stimulates their motility. LECs that express $UAS.tkv^{Q\text{-}D}$ are marked with mCD8-GFP. Cells do not die and accumulate at the dorsal midline. Furthermore, cells do not stop moving but continue to migrate posteriorly. Segments A2 and A3 shown. Image area is 303 μm^2. Frames taken from this movie and trajectory plot shown in Fig. 4G.

Supplementary Movie 10. ds-RNAi in the LECs interferes with their posterior migration. LECs that express *UAS.ds-RNAi* are marked with mCD8-GFP. In pupae, in which most LECs express *UAS.ds-RNAi*, the net posterior movement of the LECs is neutralised because of the lack of directed posterior movement of individual LECs. Two lines along the segment boundaries help to appreciate this lack of posterior movement. Note that the dorsal migrations appear unaffected – cells move straight towards the midline. Segment A2 shown. Image area is 218 by 262 μm.

Supplementary Movie 11. Overexpression of a constitutively active form of Rho1 drives LECs to constrict without posterior migration. One hemisegment of segment A2 shown. Clones of LECs overexpressing $UAS.rho^{V14}$ are marked with RFP. All cells express DE-cadherin::GFP. Left: GFP-channel; right: merge of GFP- and RFP-channels. LECs that express $UAS.rho^{V14}$ from the start of the movie constrict without any cell shape change and protrusive activity. LECs that begin to express RFP (and

Rho^{V14}) later in the movie start to constrict soon after the RFP expression comes up. Some LECs that start RFP expression late generate protrusions before (white arrow). Overall, no posterior migration can be observed, and the dorsal movement is likely to be solely due to constriction of the LECs. Note that those LECs that express *UAS.rho*^{V14} from the start show higher DE-cadherin::GFP levels at their junctions compared to the other LECs, even if they have the same size. LECs that start to express RFP (and thus Rho^{V14}) at a later stage increase fluorescence at their adherens junctions during the course of the movie. Image area is 203 by 337 μm. Frames from this movie and trajectory plot shown in Fig. 6A, D.

Supplementary Movie 12. Overexpression of a constitutively active form of Rho1 drives LECs towards apical constriction. LECs overexpressing *UAS.rho*^{V14} are marked with mCD8-GFP. *UAS.rho*^{V14} LECs constrict without showing migratory behaviour – they do not move posteriorly and immediately constrict all over the tissue without cell shape change (spreading) along the a-p axis. Interestingly, also the single row of LECs that persists at the segment boundary in wild-type pupae (see Supplementary Movie 1) is constricting and does hence not remain. LECs leave GFP-positive 'footprints' (red arrows), which are positioned approximately 10 μm apical to the histoblasts (a few histoblasts are also GFP-positive (white arrows)). This suggests that cells that are driven to constrict do not properly detach from their apical substrate. Interestingly, the constriction leads to an accumulation of cells at the dorsal midline, and only then extensive cell death occurs (GFP-positive fragments of dead cells can be seen). This suggests that Rho1^{V14} drives cells to constrict but does not increase the rate of cell death. Segments A2 and A3 shown. Image area is 338 by 472 μm. A single frame taken from this movie shown in Fig. 6B.

Supplementary Movie 13. Knock-down of Rho1 by RNAi leads to a spreading of the LECs. LECs overexpressing *UAS.rho1-RNAi* are marked with mCD8-GFP. *UAS.rho1-RNAi* LECs do constrict, but prior to constriction they display extensive spreading with increased apical area (one cell is highlighted with white arrows). Segments A1 to A3 shown. Segment A1 and a part of A2 move out of focus during the course of the movie. Image area is 376 μm^2. A single frame taken from this movie shown in Fig. 6C.

Supplementary Movie 14. $Rho1^{N19}$ overexpression in LECs eventually leads to a tearing of the epithelium, which may be caused by a reduction in cell-cell adhesion. The epithelium tears open (orange arrows in first frame) and the underlying fat body tissue becomes visible. During the course of the movie, two more areas tear open (orange arrows and dotted lines). The LECs that overexpress $Rho1^{N19}$ are marked with mCD8-GFP. See also Supplementary Fig. 3B.

Supplementary Movie 15. The DE-cadherin-GFP signal at the adherens junctions of wild-type LECs gets weaker during the transition from stationary to migratory behaviour. For this movie, Supplementary Movie 2 has been coloured using the 'fire' look-up table (ImageJ, Bethesda, USA) to highlight the change in GFP-signal intensity. 150 min into the movie, three LECs are highlighted in the left box, while the right box shows the same three LECs at the start of the movie, which illustrates the decrease in fluorescence at the adherens junctions. The reduction in fluorescence intensity appears not to be due to photo-bleaching, as it is not linear throughout the course of the movie. The development of a hemisegment of segment A2 is shown.

The dorsal midline is located just outside the top of the image. DE-cadherin::GFP labels all membranes. Image area is 244 μm^2.

Supplementary References

Bloor, J.W., Kiehart, D.P., 2001. zipper Nonmuscle myosin-II functions downstream of PS2 integrin in *Drosophila* myogenesis and is necessary for myofibril formation. Dev. Biol. 239**,** 215-28.

Dietzl, G., Chen, D., Schnorrer, F., Su, K. C., Barinova, Y., Fellner, M., Gasser, B., Kinsey, K., Oppel, S., Scheiblauer, S., Couto, A., Marra, V., Keleman, K., Dickson, B.J., 2007. A genome-wide transgenic RNAi library for conditional gene inactivation in *Drosophila*. Nature. 448**,** 151-6.

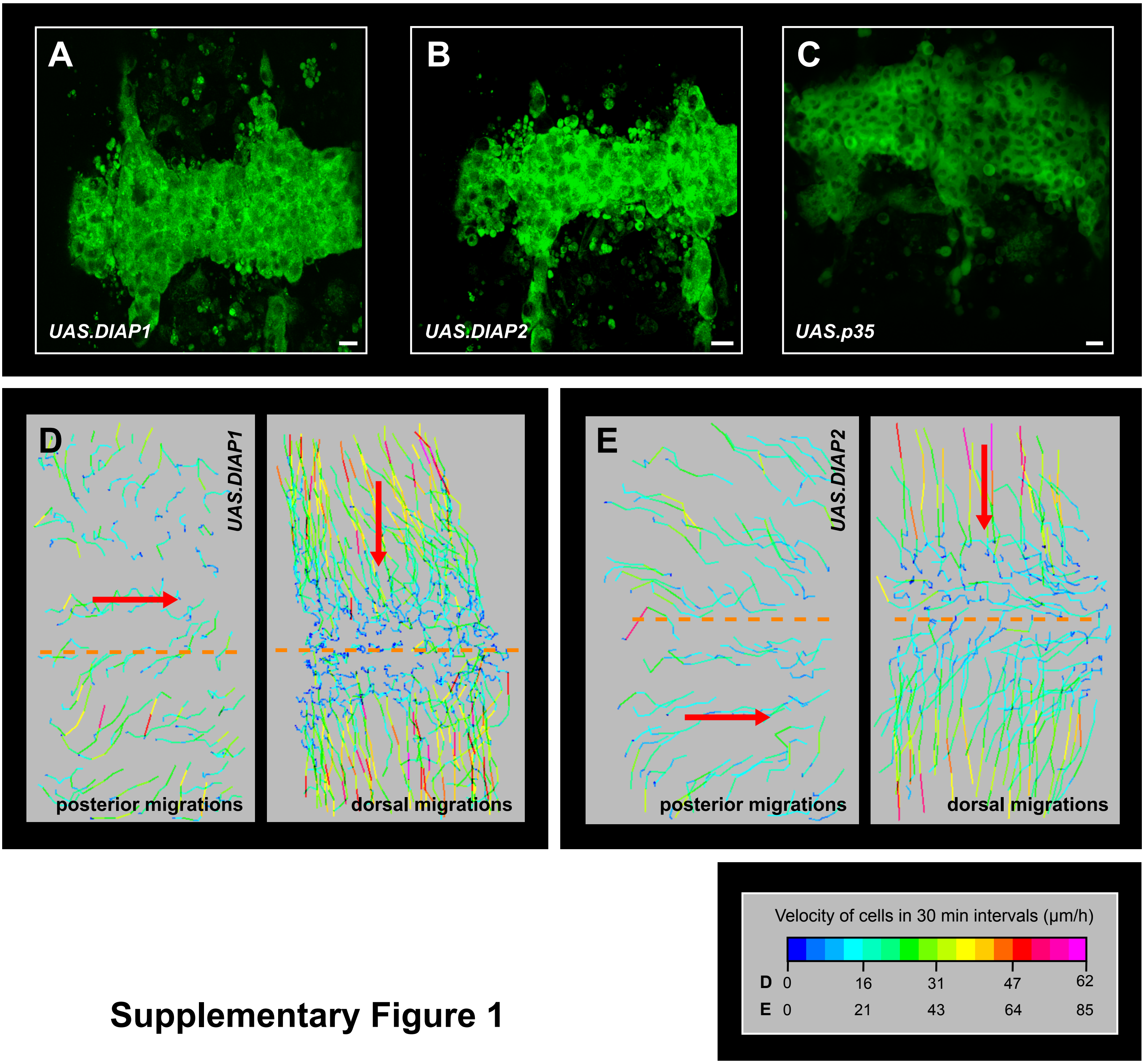

Supplementary Figure 1

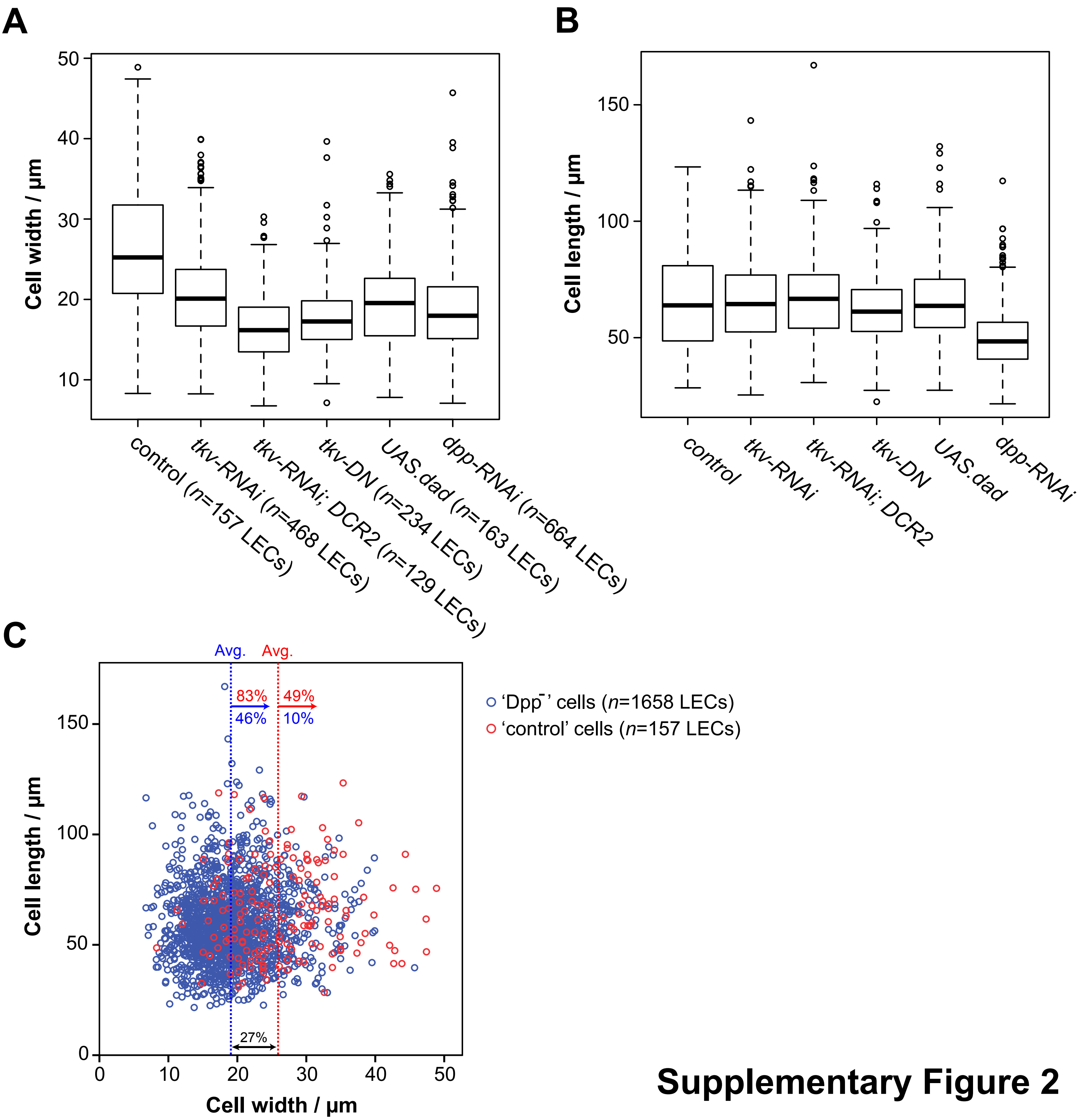

Supplementary Figure 2

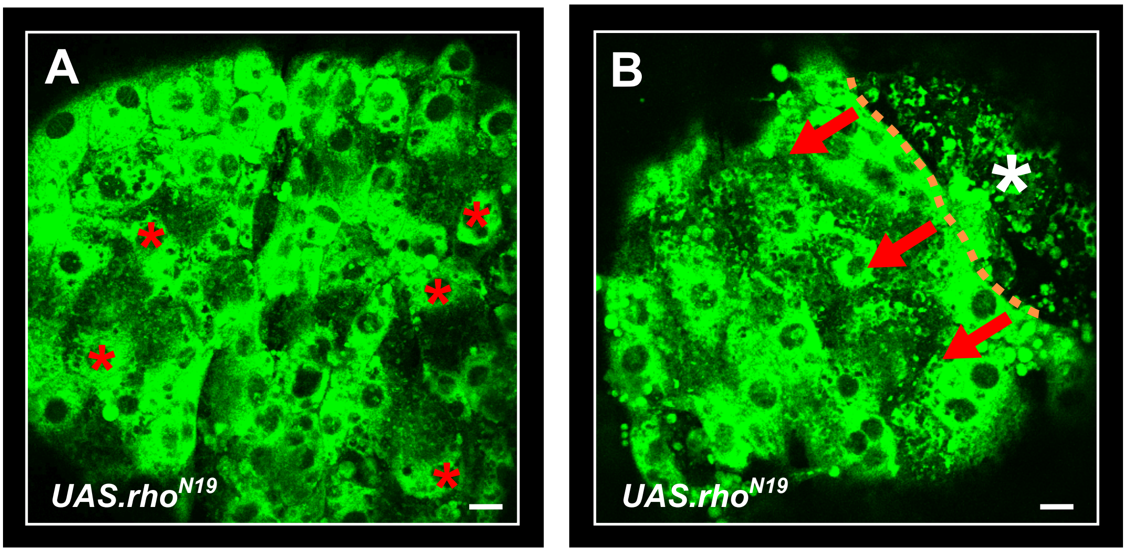

Supplementary Figure 3

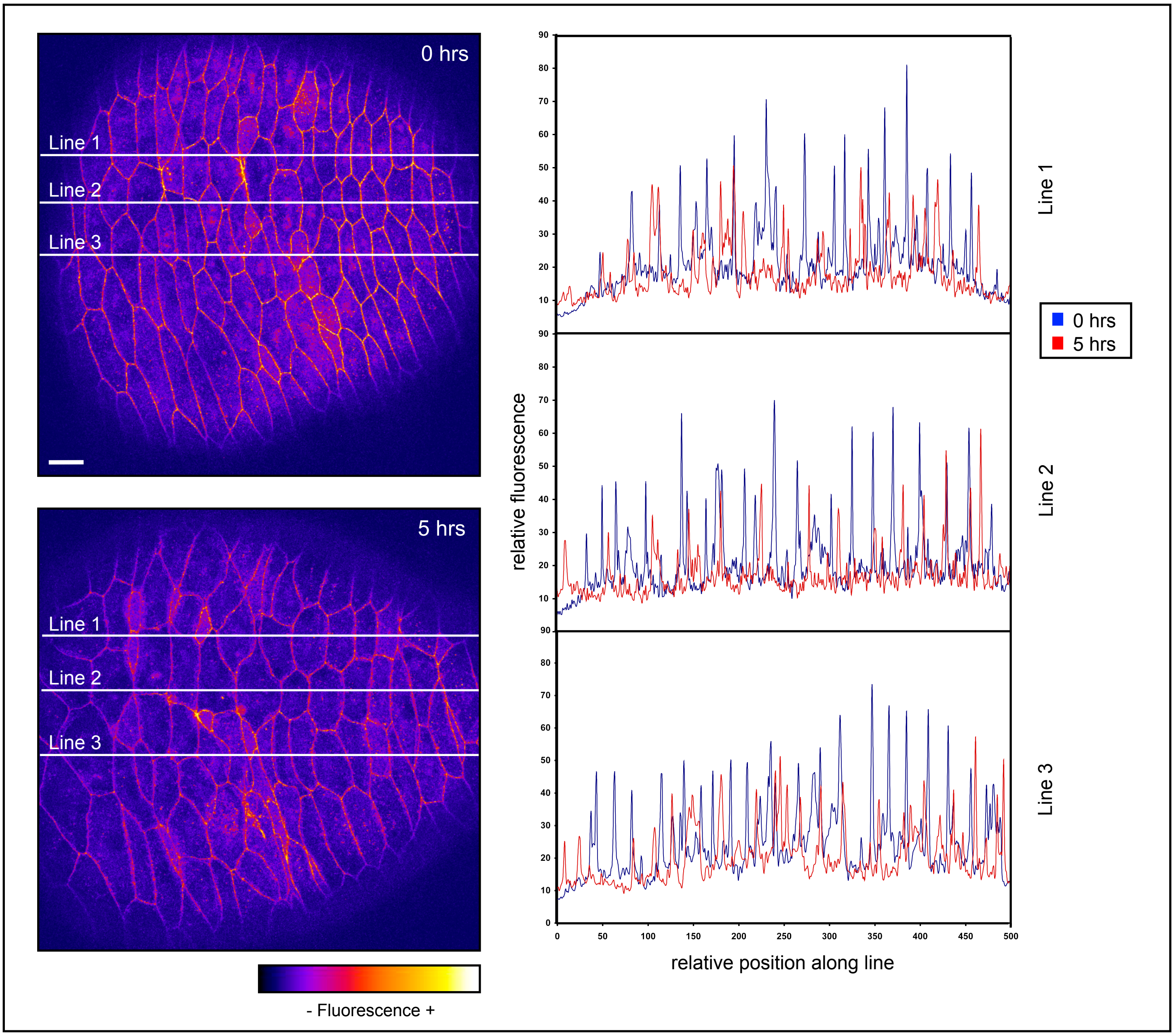

Supplementary Figure 4

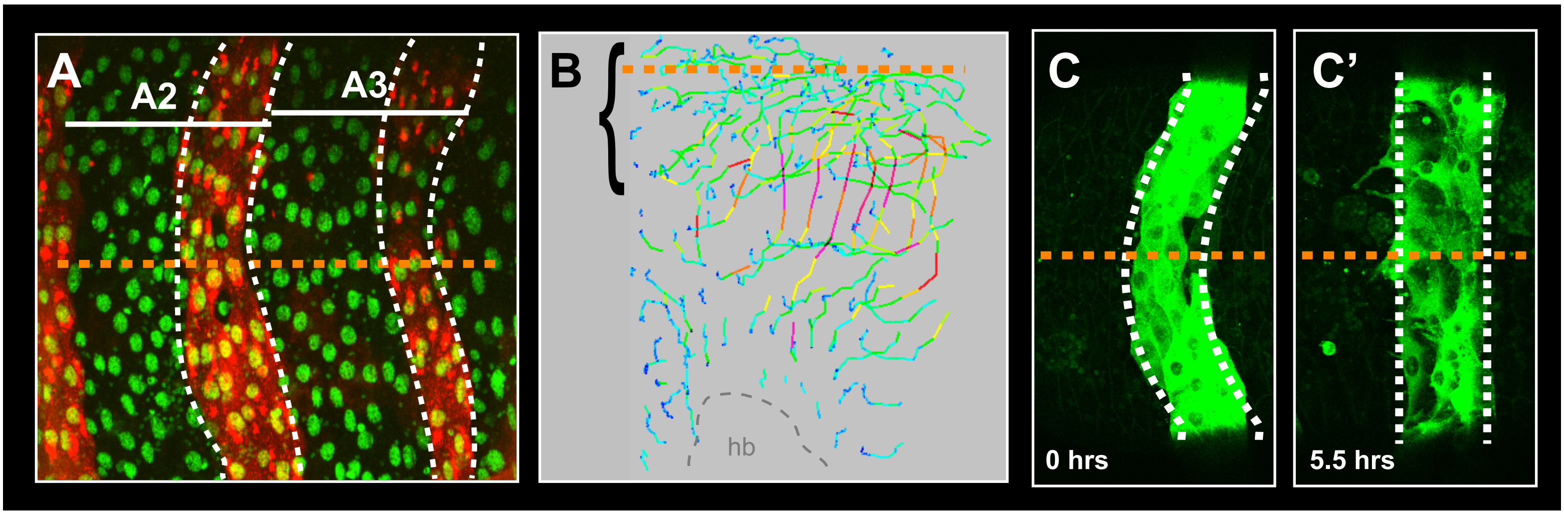

Supplementary Figure 5

Supplementary Table 1. *n*-numbers of recorded pupae per experiment.

Genotype	*n*
H2AvGFP[1]	3
DE-cadherin::GFP	4
UAS.mCD8-GFP	13
UAS.gma	7
UAS.pio-RNAi	5
UAS.p35	7
UAS.DIAP1	3
UAS.DIAP2	4
UAS.tkv$^{Q\text{-}D}$	5
UAS.ds-RNAi	14
UAS.ectoDs	6
UAS.rho^{V14} *(DE-cadherin-GFP)*	3
UAS.rho^{V14} *(mCD8-GFP)*	6
UAS.rho1-RNAi	6
UAS.rho^{N19} *(DE-cadherin-GFP)*	3
UAS.rho^{N19} *(mCD8-GFP)*	3

[1] see also Bischoff and Cseresnyes (2009)
For other Dpp signalling experiments see Supplementary Table 2.

Supplementary Table 2. Manipulation of Dpp signalling affects different aspects of cell motility.

Genotype	(1) Posterior migration absent[1]	(2) Cell shape change absent (% of these pupae that have narrow cells)	(3) Dorsal migration drifts posteriorly	*n*
UAS.tkv-RNAi	26%	100% (93%)	71%	14
UAS.tkv-RNAi; UAS.DCR2	33%	100% (67%)	67%	3
UAS.tkv^{DN}	0%	100% (72%)	100%	7
UAS.dad	60%	100% (80%)	40%	5
UAS.dpp-RNAi	9%	36%[2] (25%)	73%	11

[1] LECs move less than one cell diameter.
[2] In another 36% of the pupae, most cells display shape changes but a few individual cells remain elongated along the d-v axis.
