# Supplementary figures and images for: Lamellipodia-based migrations of larval epithelial cells are required for normal closure of the adult epidermis of Drosophila
Source: Dev Biol. 2012 Mar 1;363-135(1):179–90. doi: 10.1016/j.ydbio.2011.12.033 (PMC3314956; doi:10.1016/j.ydbio.2011.12.033)

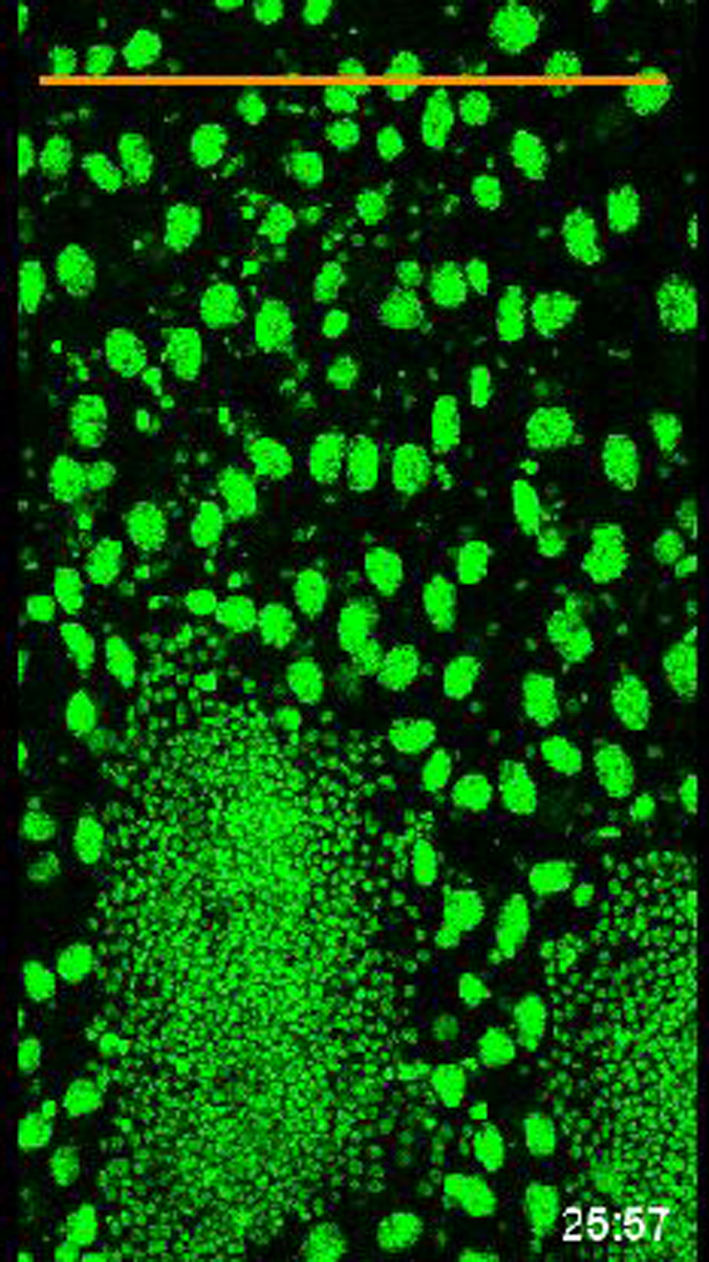

Supplement: Supplementary Movie 1 — Wild-type development of segment A2. A Histone::GFP marker marks all nuclei. The histoblasts (small nuclei) move towards the midline and replace the LECs (large nuclei). One row of LECs that delaminate later separates the histoblasts of neighbouring segments laterally from each other. Image area is 275 by 514 μm. See Fig. 1 C for trajectory plot. [file mmc2.jpg]

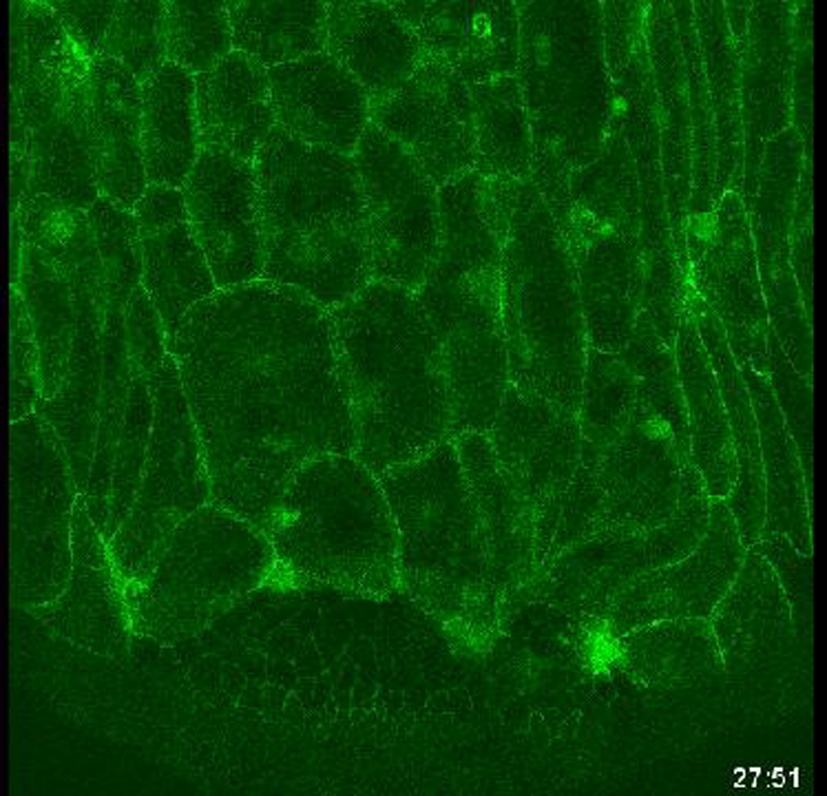

Supplement: Supplementary Movie 2 — Cell shape changes and migration of the LECs. The development of a hemisegment of segment A2 is shown. The dorsal midline is located outside the top of the image. DE-cadherin::GFP labels all membranes. The small histoblasts replace the big LECs. Before the histoblasts move into the image from the bottom and the LECs start to move in posterior direction, the LECs change their shape. This cell shape change mainly occurs along the a–p axis (green arrow). During posterior migration, cells display crescent-shaped protrusions, which point posteriorly (white arrows). During dorsal migration, the protrusions point in dorsal direction (blue arrows). During the whole process, LECs constrict apically, delaminate and die (red arrow). However, constriction is most extensive while LECs are moving dorsally. Image area is 244 μm2. Frames from this movie shown in Figs. 1A, D. [file mmc3.jpg]

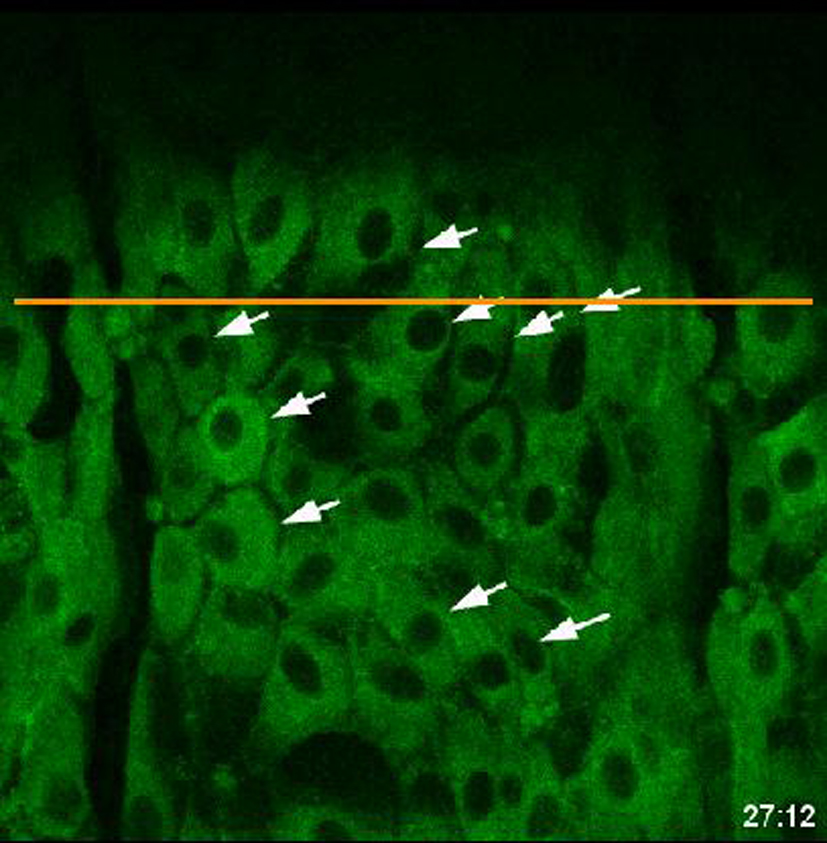

Supplement: Supplementary Movie 3 — Wild-type development. LECs are marked with mCD8-GFP. When LECs undergo the transition from stationary to migratory behaviour, they change shape mainly along the anterior–posterior axis (green arrow). Then cells migrate posteriorly and subsequently dorsally, displaying apical lamellipodia-like protrusions in posterior (white arrows) and dorsal (red arrows) direction, respectively. Note that the cells do not lose contact (see Supplementary Movie 2) — the areas that appear darker only do so because the spreading cells get thinner. Some LECs die before migrations begin. Segment A2 and parts of the neighbouring segments shown. The right hemisegment is moving out of focus in the course of the movie. Image area is 304 μm2. hb, histoblasts, which are GFP-negative. Wild-type development. LECs are marked with mCD8-GFP. When LECs undergo the transition from stationary to migratory behaviour, they change shape mainly along the anterior–posterior axis (green arrow). Then cells migrate posteriorly and subsequently dorsally, displaying apical lamellipodia-like protrusions in posterior (white arrows) and dorsal (red arrows) direction, respectively. Note that the cells do not lose contact (see Supplementary Movie 2) — the areas that appear darker only do so because the spreading cells get thinner. Some LECs die before migrations begin. Segment A2 and parts of the neighbouring segments shown. The right hemisegment is moving out of focus in the course of the movie. Image area is 304 μm2. hb, histoblasts, which are GFP-negative. [file mmc4.jpg]

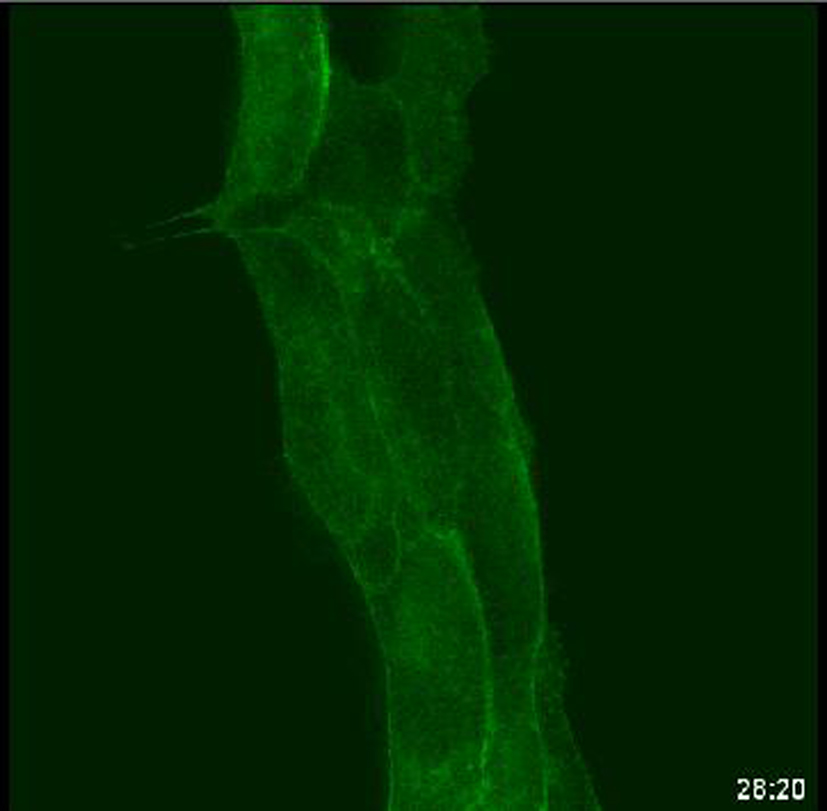

Supplement: Supplementary Movie 4 — Posterior migration of the LECs. LECs of the P compartment are labelled with UAS.gma (Bloor and Kiehart, 2001) driven by hh.Gal4. GMA is an actin-binding fragment of moesin fused with GFP, which labels the actin cytoskeleton. LECs move posteriorly, displaying crescent-shaped lamellipodia-like protrusions (blue arrows). Note that all protrusions point posteriorly and that most protrusions are positioned ‘on top’ of a neighbouring, more posterior cell (see Fig. 2 C). At the posterior boundary of the segment, the protrusions can be seen extending towards the unlabelled neighbours (white arrows). Some LECs produce retraction fibres at their back (green arrows). Some cells delaminate (red arrow). Image area is 206 μm2. A frame taken from this movie shown in Fig. 2 C. [file mmc5.jpg]

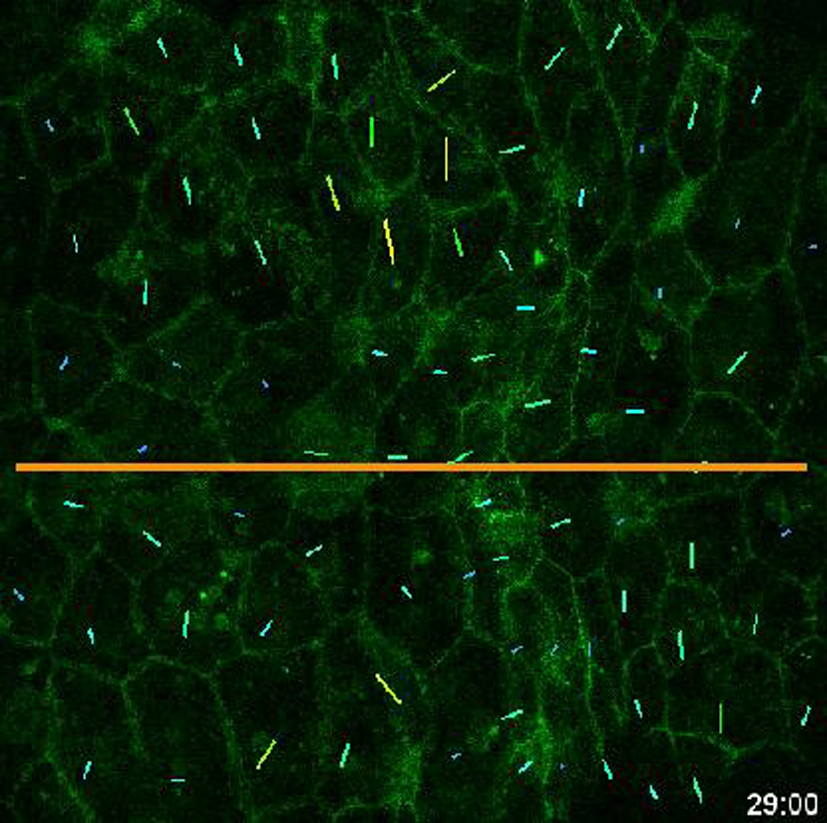

Supplement: Supplementary Movie 5 — LECs generate protrusions in the direction of movement. In many cases, the protrusions of the LECs point in the direction of movement. At later stages, cells stop migrating and merely constrict apically. The paths the LECs move in 30 min intervals are merged with confocal images. Line colour indicates the speed of the LECs as shown in Fig. 2. All cells are marked with DE-cadherin::GFP. Image area is 356 μm2. A frame taken from this movie shown in Fig. 2D. [file mmc6.jpg]

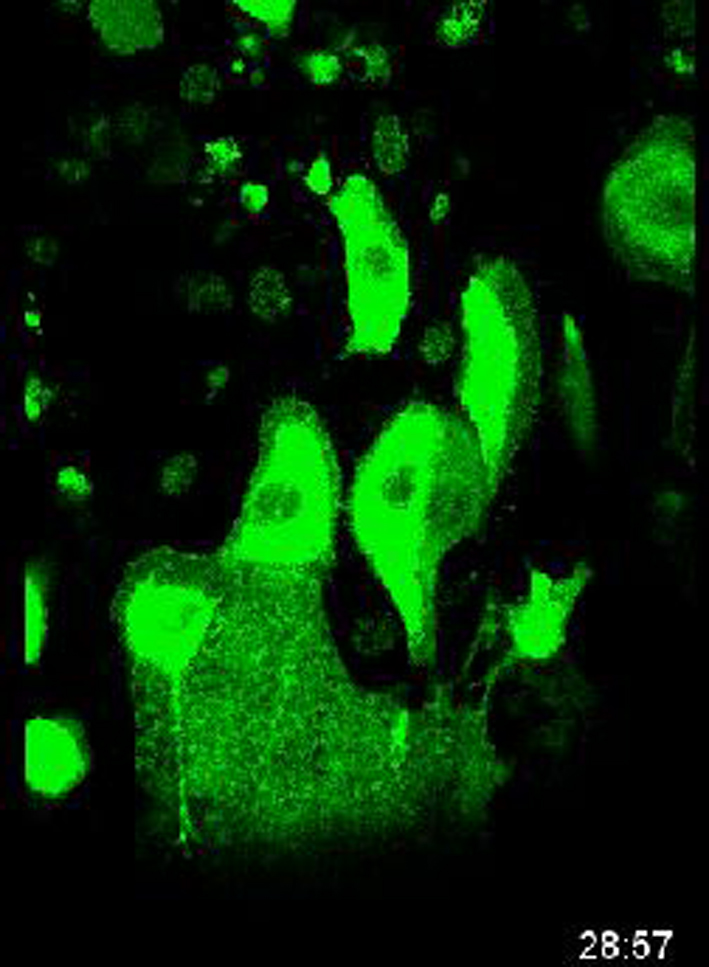

Supplement: Supplementary Movie 6 — LECs repolarise when approached by the histoblasts. mCD8-GFP marks membranes of clones of LECs in a hemisegment of segment A2. At the beginning of the movie, the histoblasts move into the image from the bottom (hb). Please focus on the LEC marked with an asterisk. It begins to produce a lamellipodia-like protrusion in posterior direction (cyan arrows), but when approached by the histoblasts (white arrow), it repolarises in dorsal direction. Blue arrows point at a posteriorly directed protrusion of another LEC. When migrating dorsally, the LECs generate dorsally directed protrusions (red arrows). Note that cells constrict while they are moving dorsally. Image area is 237 by 348 μm. Frames taken from this movie shown in Fig. 2E. [file mmc7.jpg]

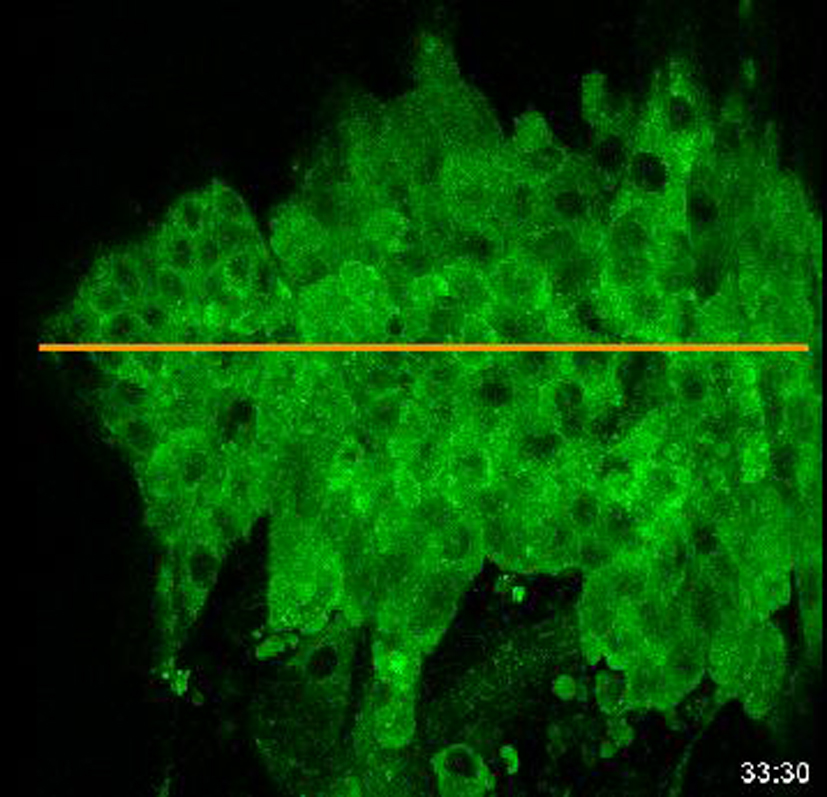

Supplement: Supplementary Movie 7 — LECs overexpressing DIAP2 migrate normally. LECs that overexpress UAS.DIAP2 are marked with mCD8-GFP. Segments A1 to A3 shown. LECs move posteriorly and then dorsally, although persisting LECs seem to limit the space for other cells to move, leading to ‘traffic jams’. Since cells do not delaminate, many LECs remain at the dorsal midline, resulting in a dorsal closure defect. Image area is 367 μm2. A single frame from this movie and trajectory plot shown in Supplementary Figs. 1B, E. [file mmc8.jpg]

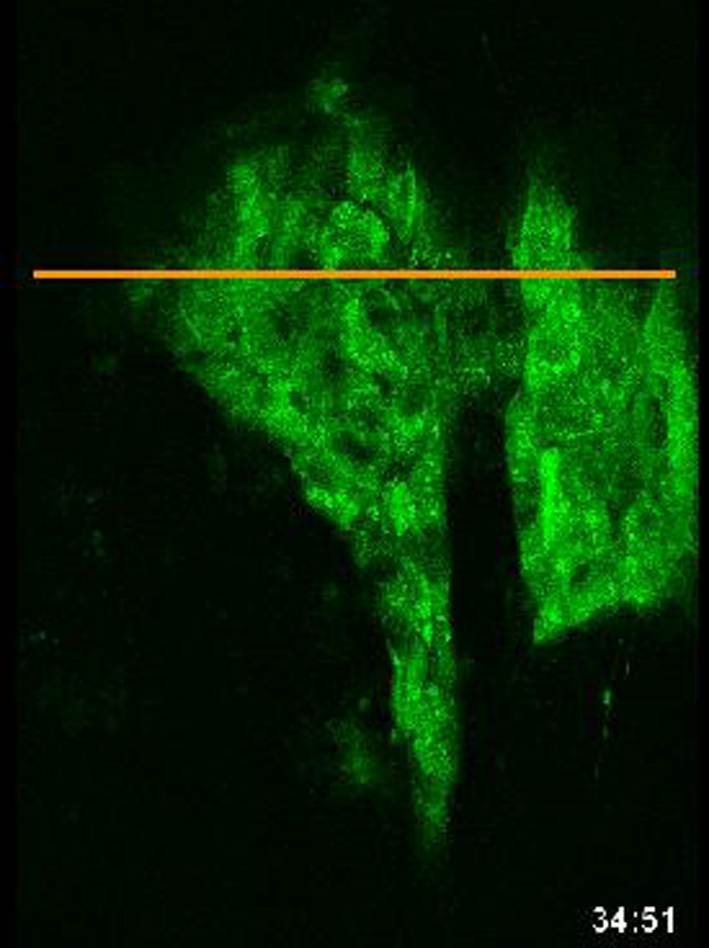

Supplement: Supplementary Movie 8 — Overexpression of Dad impairs cell motility. LECs that express UAS.dad are marked with mCD8-GFP. Cells do not spread in a–p direction and appear elongated in d–v direction (white bars). Posterior migration is virtually absent. Cells close to the histoblasts become round and drift dorsally (white arrows). Segments A2 and A3 shown. Image area is 283 by 395 μm. A single frame taken from this movie and trajectory plot shown in Fig. 4D. [file mmc9.jpg]

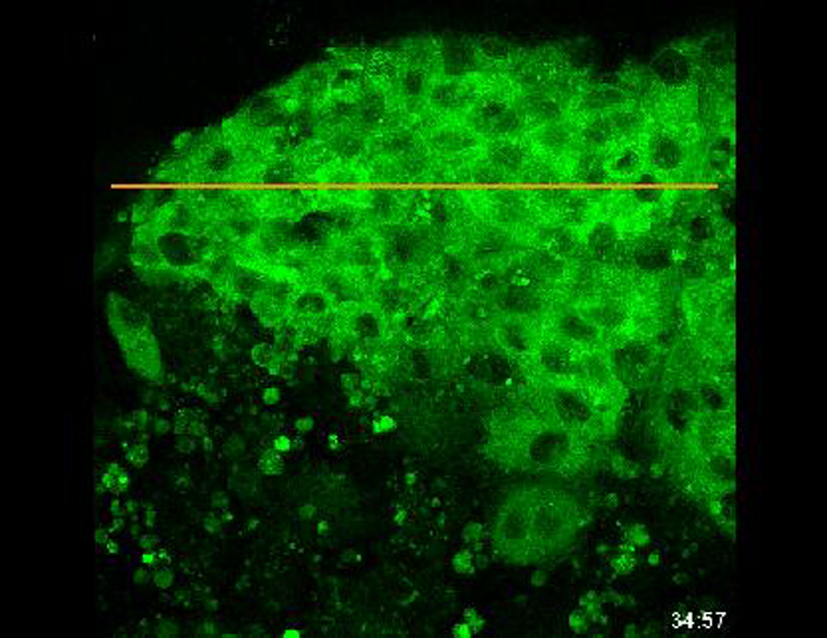

Supplement: Supplementary Movie 9 — Overexpression of a constitutively active form of Tkv in LECs stimulates their motility. LECs that express UAS.tkvQ-D are marked with mCD8-GFP. Cells do not die and accumulate at the dorsal midline. Furthermore, cells do not stop moving but continue to migrate posteriorly. Segments A2 and A3 shown. Image area is 303 μm2. Frames taken from this movie and trajectory plot shown in Fig. 4G. [file mmc10.jpg]

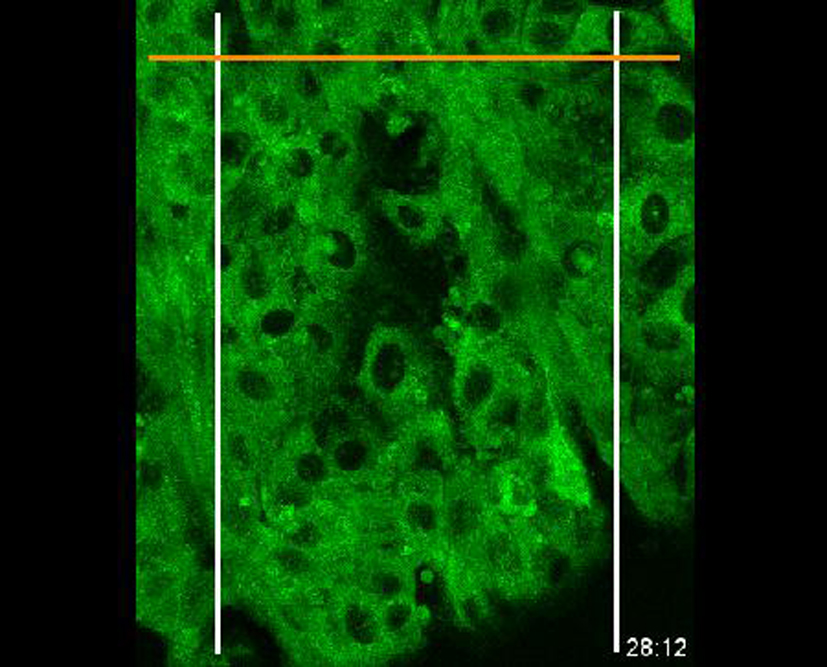

Supplement: Supplementary Movie 10 — ds-RNAi in the LECs interferes with their posterior migration. LECs that express UAS.ds-RNAi are marked with mCD8-GFP. In pupae, in which most LECs express UAS.ds-RNAi, the net posterior movement of the LECs is neutralised because of the lack of directed posterior movement of individual LECs. Two lines along the segment boundaries help to appreciate this lack of posterior movement. Note that the dorsal migrations appear unaffected — cells move straight towards the midline. Segment A2 shown. Image area is 218 by 262 μm. [file mmc11.jpg]

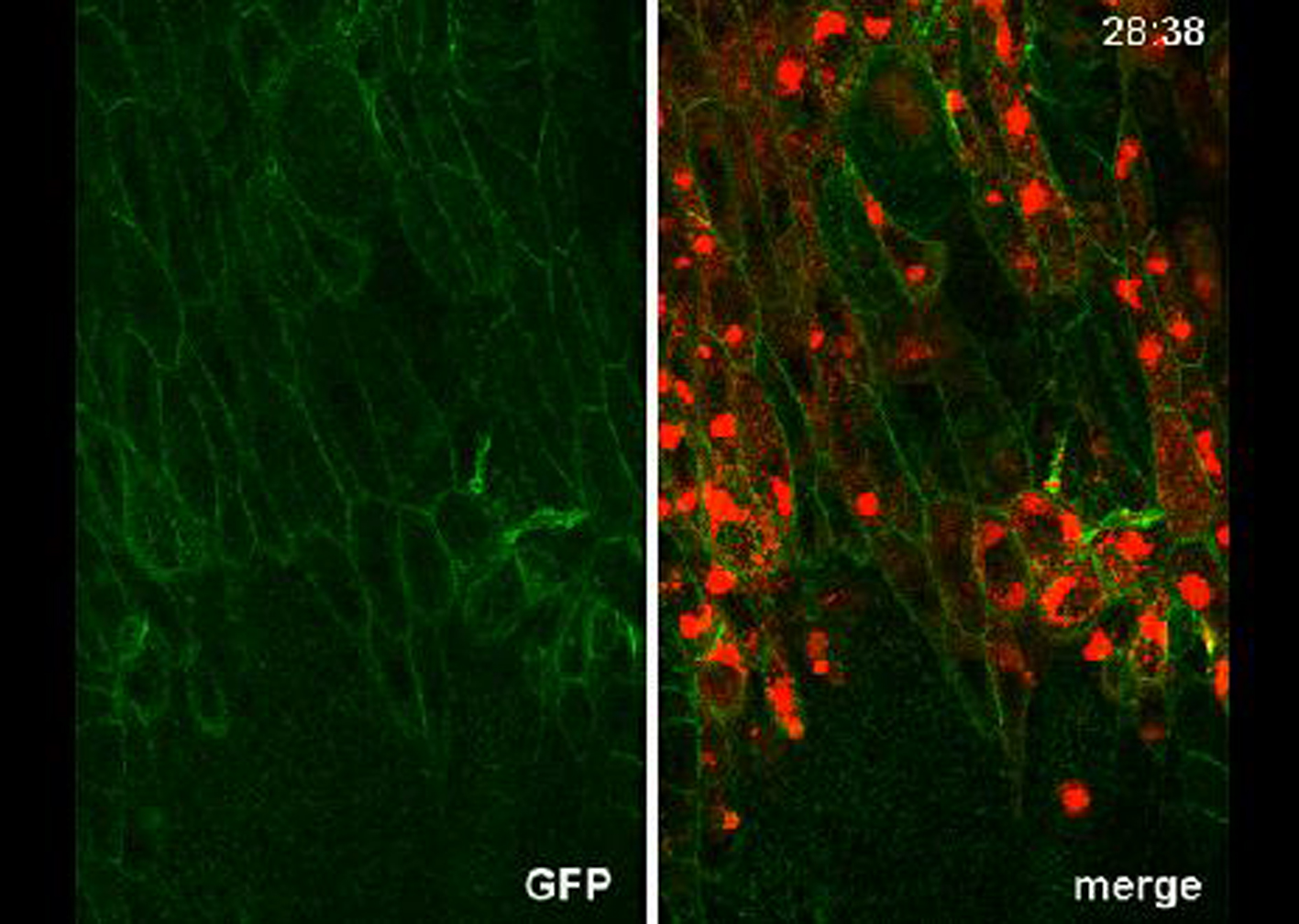

Supplement: Supplementary Movie 11 — Overexpression of a constitutively active form of Rho1 drives LECs to constrict without posterior migration. One hemisegment of segment A2 shown. Clones of LECs overexpressing UAS.rhoV14 are marked with RFP. All cells express DE-cadherin::GFP. Left: GFP-channel; right: merge of GFP- and RFPchannels. LECs that express UAS.rhoV14 from the start of the movie constrict without any cell shape change and protrusive activity. LECs that begin to express RFP (and RhoV14) later in the movie start to constrict soon after the RFP expression comes up. Some LECs that start RFP expression late generate protrusions before (white arrow). Overall, no posterior migration can be observed, and the dorsal movement is likely to be solely due to constriction of the LECs. Note that those LECs that express UAS.rhoV14 from the start show higher DE-cadherin::GFP levels at their junctions compared to the other LECs, even if they have the same size. LECs that start to express RFP (and thus RhoV14) at a later stage increase fluorescence at their adherens junctions during the course of the movie. Image area is 203 by 337 μm. Frames from this movie and trajectory plot shown in Figs. 6A, D. [file mmc12.jpg]

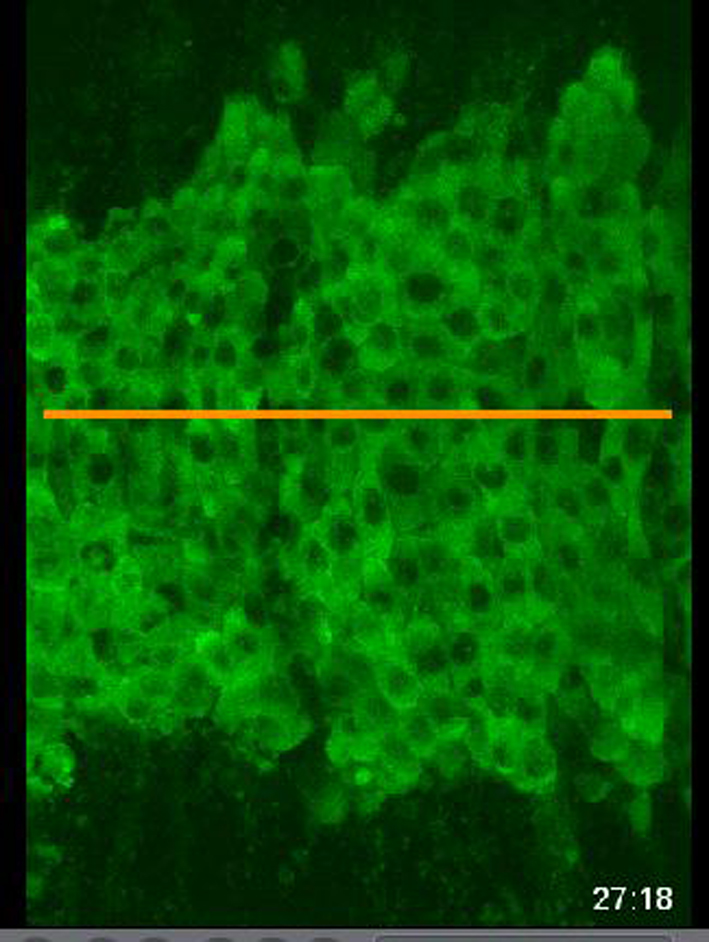

Supplement: Supplementary Movie 12 — Overexpression of a constitutively active form of Rho1 drives LECs towards apical constriction. LECs overexpressing UAS.rhoV14 are marked with mCD8-GFP. UAS.rhoV14 LECs constrict without showing migratory behaviour — they do not move posteriorly and immediately constrict all over the tissue without cell shape change (spreading) along the a–p axis. Interestingly, also the single row of LECs that persists at the segment boundary in wild-type pupae (see Supplementary Movie 1) is constricting and does hence not remain. LECs leave GFP-positive ‘footprints’ (red arrows), which are positioned approximately 10 μm apical to the histoblasts (a few histoblasts are also GFP-positive (white arrows)). This suggests that cells that are driven to constrict do not properly detach from their apical substrate. Interestingly, the constriction leads to an accumulation of cells at the dorsal midline, and only then extensive cell death occurs (GFP-positive fragments of dead cells can be seen). This suggests that RhoV14 drives cells to constrict but does not increase the rate of cell death. Segments A2 and A3 shown. Image area is 338 by 472 μm. A single frame taken from this movie shown in Fig. 6B. Overexpression of a constitutively active form of Rho1 drives LECs towards apical constriction. LECs overexpressing UAS.rhoV14 are marked with mCD8-GFP. UAS.rhoV14 LECs constrict without showing migratory behaviour — they do not move posteriorly and immediately constrict all over the tissue without cell shape change (spreading) along the a–p axis. Interestingly, also the single row of LECs that persists at the segment boundary in wild-type pupae (see Supplementary Movie 1) is constricting and does hence not remain. LECs leave GFP-positive ‘footprints’ (red arrows), which are positioned approximately 10 μm apical to the histoblasts (a few histoblasts are also GFP-positive (white arrows)). This suggests that cells that are driven to constrict do not properly detach from their apical substrate. Int [file mmc13.jpg]

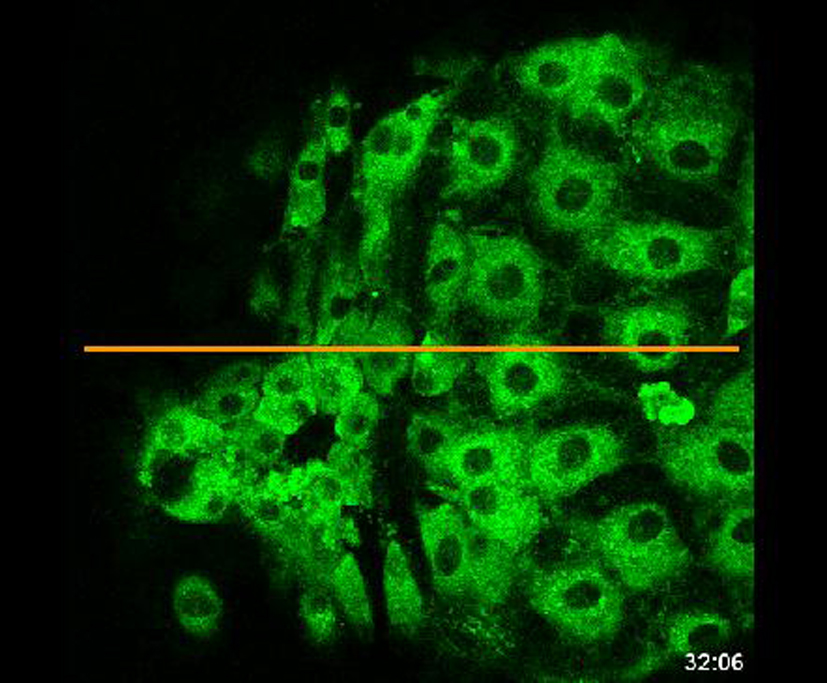

Supplement: Supplementary Movie 13 — Knock-down of Rho1 by RNAi leads to a spreading of the LECs. LECs overexpressing UAS.rho1-RNAi are marked with mCD8-GFP. UAS.rho1-RNAi LECs do constrict, but prior to constriction they display extensive spreading with increased apical area (one cell is highlighted with white arrows). Segments A1 to A3 shown. Segment A1 and a part of A2 move out of focus during the course of the movie. Image area is 376 μm2. A single frame taken from this movie shown in Fig. 6C. [file mmc14.jpg]

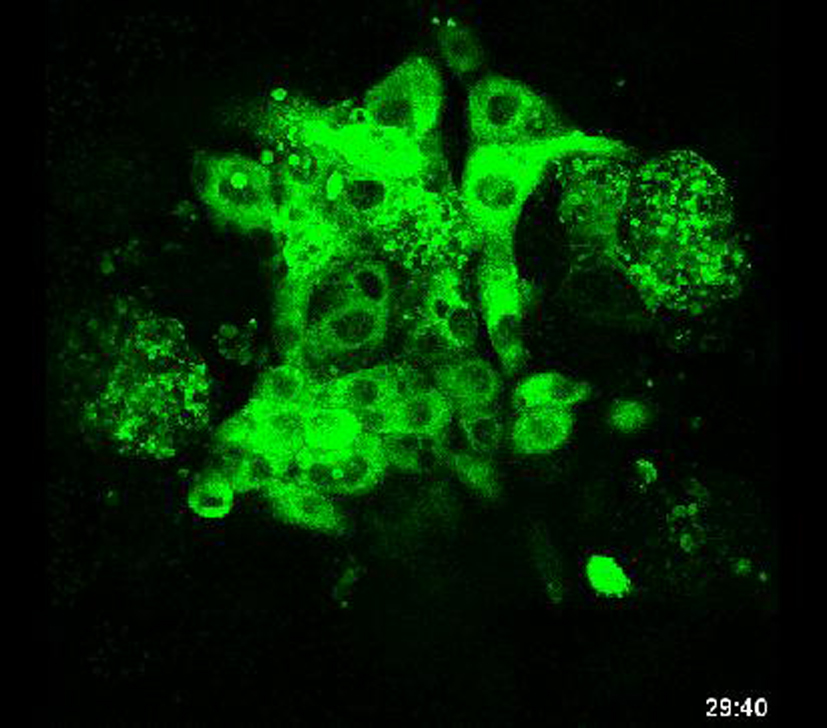

Supplement: Supplementary Movie 14 — Rho1N19 overexpression in LECs eventually leads to a tearing of the epithelium, which may be caused by a reduction in cell–cell adhesion. The epithelium tears open (orange arrows in first frame) and the underlying fat body tissue becomes visible. During the course of the movie, two more areas tear open (orange arrows and dotted lines). The LECs that overexpress Rho1N19 are marked with mCD8-GFP. See also Supplementary Fig. 3B. Rho1N19 overexpression in LECs eventually leads to a tearing of the epithelium, which may be caused by a reduction in cell–cell adhesion. The epithelium tears open (orange arrows in first frame) and the underlying fat body tissue becomes visible. During the course of the movie, two more areas tear open (orange arrows and dotted lines). The LECs that overexpress Rho1N19 are marked with mCD8-GFP. See also Supplementary Fig. 3B. [file mmc15.jpg]
